# Comparative Phenotypic, Proteomic, and Phosphoproteomic Analysis Reveals Different Roles of Serine/Threonine Phosphatase and Kinase in the Growth, Cell Division, and Pathogenicity of *Streptococcus suis*

**DOI:** 10.3390/microorganisms9122442

**Published:** 2021-11-26

**Authors:** Qiao Hu, Lun Yao, Xia Liao, Liang-Sheng Zhang, Hao-Tian Li, Ting-Ting Li, Qing-Gen Jiang, Mei-Fang Tan, Lu Li, Roger R. Draheim, Qi Huang, Rui Zhou

**Affiliations:** 1 State Key Laboratory of Agricultural Microbiology, College of Veterinary Medicine, Huazhong Agricultural University, Wuhan 430070, China; huqiao@webmail.hzau.edu.cn (Q.H.); yaolun@webmail.hzau.edu.cn (L.Y.); lx287486056@163.com (X.L.); zls2015@webmail.hzau.edu.cn (L.-S.Z.); lht@webmail.hzau.edu.cn (H.-T.L.); li.tingting@webmail.hzau.edu.cn (T.-T.L.); jiangqinggen@webmail.hzau.edu.cn (Q.-G.J.); lilu@mail.hzau.edu.cn (L.L.); 2 Institute of Animal Husbandry and Veterinary Science, Jiangxi Academy of Agricultural Sciences, Nanchang 330200, China; missphd@126.com; 3 Cooperative Innovation Center of Sustainable Pig Production, Wuhan 430070, China; 4 International Research Center for Animal Disease, Ministry of Science and Technology of China, Wuhan 430070, China; 5 School of Pharmacy and Biomedical Sciences, University of Portsmouth, Portsmouth PO1 2UP, UK; roger.draheim@port.ac.uk

**Keywords:** *Streptococcus suis*, serine/threonine phosphatase, serine/threonine kinase, coordinated regulation, growth, cell division, pathogenicity

## Abstract

Eukaryote-like serine/threonine kinases (STKs) and cognate phosphatases (STPs) comprise an important regulatory system in many bacterial pathogens. The complexity of this regulatory system has not been fully understood due to the presence of multiple STKs/STPs in many bacteria and their multiple substrates involved in many different physiological and pathogenetic processes. *Streptococci* are the best materials for the study due to a single copy of the gene encoding STK and its cognate STP. Although several studies have been done to investigate the roles of STK and STP in zoonotic *Streptococcus suis*, respectively, few studies were performed on the coordinated regulatory roles of this system. In this study, we carried out a systemic study on STK/STP in *S. suis* by using a comparative phenotypic, proteomic, and phosphoproteomic analysis. Mouse infection assays revealed that STK played a much more important role in *S. suis* pathogenesis than STP. The ∆*stk* and ∆*stp*∆*stk* strains, but not ∆*stp*, showed severe growth retardation. Moreover, both ∆*stp* and ∆*stk* strains displayed defects in cell division, but they were abnormal in different ways. The comparative proteomics and phosphoproteomics revealed that deletion of *stk* or *stp* had a significant influence on protein expression. Interestingly, more virulence factors were found to be downregulated in ∆*stk* than ∆*stp*. In ∆*stk* strain, a substantial number of the proteins with a reduced phosphorylation level were involved in cell division, energy metabolism, and protein translation. However, only a few proteins showed increased phosphorylation in ∆*stp*, which also included some proteins related to cell division. Collectively, our results show that both STP and STK are critical regulatory proteins for *S. suis* and that STK seems to play more important roles in growth, cell division, and pathogenesis.

## 1. Introduction

Reversible protein phosphorylation is a key mechanism that regulates many cellular processes in both prokaryotes and eukaryotes [1]. In bacteria, protein phosphorylation, which is mainly mediated by histidine kinases (HKs), eukaryote-like serine/threonine kinases (STKs), and tyrosine kinases, plays an important role in a variety of biological processes [2,3,4,5]. Recently, eukaryote-like serine/threonine kinases (STKs) and cognate phosphatases (STPs), as functional pairs [6,7], have emerged as important regulators with parallel or overlapping signaling networks for the regulation of many different physiological and pathogenetic processes in bacteria [3,8].

STK plays an important role in many aspects, including growth, metabolism, biofilm formation, stress response, adhesion, and pathogenesis in many pathogenic bacteria, such as *Mycobacteria tuberculosis* [9,10], *Staphylococcus aureus* [11,12], *Streptococcus pneumoniae* [13,14], *Streptococcus pyogenes* [15,16], and *Streptococcus suis* [17,18,19,20,21]. STK itself acts as an important component of the cell division machinery. In *S. pneumoniae*, STK localizes to division sites, resulting in elongated morphologies when it is deleted [22]. The extracellular PASTA repeats of STK are required for regulating its autophosphorylation and septal cell wall synthesis [23]. In addition, STK influences a wide range of physiological processes through its substrate proteins via modulating their phosphorylation states. The verified or suspected substrates of STK include DivIVA [24,25], MapZ [26], GpsB [27], FtsA, FtsZ [28], MacP [29], GlmM [7,30], and MurC [31].

STP is a negative regulator of kinase activity and a global serine/threonine phosphatase [32]. Unlike STK, the physiological roles of STP are less well understood. Previous studies have suggested that STPs are involved in the regulation of bacterial cell wall synthesis [33], cell division [32,34], and virulence [35]. However, STP is not essential for the growth or survival of the encapsulated *S. pneumoniae* D39 strain [36]. Proteomic analysis has revealed that cell division-related proteins, including DivIVA, MapZ, GpsB, MreC, MltG, GlmM, MacP, and Jag could be its potential substrates [8], but further studies and direct evidence are needed to reveal more substrates of bacterial STPs.

The complexity of the STK/STP regulatory system is not yet fully understood, largely due to multiple STKs and STPs existing in many bacteria, and their multiple substrates involved in many different physiological and pathogenetic processes. For example, *M. tuberculosis* has 11 STKs [37], and *Corynebacterium glutamicum* has 4 STKs [38]. *Streptococci* including *S. pneumoniae* and *S. suis*, may be the best model bacteria for studying the functions of STK and STP because they each have a single STK and its cognate STP. It should be noted that although STK and STP are cognate pairs involved in reversible protein phosphorylation, they do not function in completely overlapped processes, but have some specific functions [3,8,39]. Moreover, most previous studies have focused on only each individual protein instead of providing a holistic understanding of the functions of the kinase-phosphatase pair. For the zoonotic *S. suis*, although several studies including ours have been done to investigate the roles of STK [17,18,19,20,21,40] and STP [41,42], respectively, only one report has attempted to study the virulence-related phenotypes and possible underlying mechanisms of STK and STP by comparing the ∆*stk* and ∆*stp* with the wild type strain [21]. This report confirmed the previous finding that STK and STP reversibly modified the phosphorylation status of the cell division protein DivIVA [24], and demonstrated that *stk* deletion reduced capsule formation and survival in macrophages while *stp* deletion did the opposite, and both ∆*stk* and ∆*stp* displayed decreased survival in whole blood and virulence in mice. In this study, we systemically compared the phenotypes of three mutant strains ∆*stk*, ∆*stp*, and ∆*stk*∆*stp* with the wild-type strain of *S. suis*, and tried to reveal the mode of the coordinated action of this regulatory system by comparative proteomic and phosphoproteomic analysis.

## 2. Materials and Methods

### 2.1. Bacterial Strains, Media, and Growth Conditions

All strains used in this study are listed in Table S1. *S. suis* was isolated from a sick pig during an epidemic outbreak in Sichuan Province, China in 2005 [43]. *S. suis* and its derivatives were cultured in tryptic soy broth (TSB) (Becton Dickinson, Sparks, NV, USA) supplemented with 10% inactivated newborn bovine serum (NBS) (Sijiqing, Hangzhou, China) at 37 °C. Erythromycin was used at a final concentration of 90 μg/mL. Chemically defined minimal medium (CDM) supplemented with 1% glucose was prepared as described previously [44].

### 2.2. Construction of Plasmids, Mutants, and Complemented Strains

All primers used in this study are listed in Table S2. Because *stp* overlaps with *stk*, to avoid affecting *stk* when deleting *stp*, the coding sequence of the erythromycin resistance gene was used to replace the CDS of *stp* [36]. Briefly, upstream and downstream 1000 bp fragments flanking *stp* and/or *stk* were amplified from the *S. suis* genome using primers Pup_F/R/Pdown_F/R, Kup_F/R/Kdown_F/R, and pKup_F/R/pKdown_F/R, and the *erm* cassette was amplified from plasmid pAT18 [45] using primers Erm_F/R. The fragments were cloned into plasmid pSET4s [46] by seamless cloning using the ClonExpress MultiS One Step Cloning Kit (Vazyme, Nanjing, China), generating the plasmids of pSET4s-P/K/PK. *S. suis* competent cells were transformed with pSET4s derived plasmids, and the single exchanged and mutant strains were screened as previously described [47]. Considering the variation in plasmid copy numbers, the complement strains were constructed by integrating the target gene into the chromosome [48]. The *stp*, *stk*, and *stp*/*stk* flanked with the respective upstream and downstream fragments were cloned into pSET4s, and the complement strains were constructed as described above. The expression of STP or STK in each strain was detected by real time quantitative PCR (RT-qPCR) and Western blotting.

### 2.3. RT-qPCR

To test the expression of *stp* or *stk*, total RNA was extracted from WT, ∆*stp*, and ∆*stk* strains. Briefly, RNA was isolated using the SV Total RNA Isolation System (Promega, Madison, WI, USA) according to the manufacturer’s instructions. HiScript Q Select RT SuperMix (+gDNA wiper) (Vazyme, Nanjing, China) was used to remove residual genomic DNA and synthesize cDNA according to the manufacturer’s instructions. cDNA was used as the template for RT-qPCR using the TB Green™ Premix Ex Taq™ II kit (Takara) according to the manufacturer’s instructions with an ABI 7500 HT Sequence Detection System (Applied Biosystems, Waltham, MA, USA). The expression level of the tested gene was calculated using the 2^−ΔΔCt^ method and normalized to the housekeeping gene *gapdh* of *S. suis* [49].

### 2.4. Western Blotting

To further confirm the mutant and complemented strains, we performed Western blot analysis to detect the expression of STP and STK in each strain as previously described [50]. Cells at the mid-log phase of each strain were collected, washed with PBS, and lysed by sonication. The amount of total protein loaded was normalized using a Micro BCA protein assay kit (Cwbiotech, Beijing, China), followed by Western blot analysis using anti-STP, anti-STK, or anti-GidA serum, respectively, which were produced in mice as previously described [51]. Antibody-tagged protein bands were detected by using the Western ECL Substrate Kit (Cat# 1705060, Bio-Rad, Hercules, CA, USA).

### 2.5. Animal Infection Experiments

All animal experiments were approved by the Laboratory Animal Monitoring Committee of Huazhong Agricultural University (HZAUMO-2019-074) and performed according to the recommendations in the Guide for the Care and Use of Laboratory Animals of Hubei Province, China. Six- to seven-week-old C57BL/6 mice with similar body weights were used for all animal infection experiments. Mice were randomly divided into different groups and injected intraperitoneally. The survival rate of the mice was recorded every 24 h post-infection for seven days. For the bacterial load assays, mice were euthanized at each indicated time point, and the organs were collected, weighed, homogenized in sterile saline, and plated onto TSA for cell counting. A competitive infection assay [52], an accurate and sensitive approach to determine relative virulence, was used to compare the pathogenicity of the three mutants, in which mice were infected intraperitoneally with a 1:1 mixture of the Δ*stp* and Δ*stk* (2.85 × 10^7^ CFU in total), Δ*stp* and Δ*stp*Δ*stk* (2.8 × 10^7^ CFU in total), Δ*stk* and Δ*stp*Δ*stk* (3.05 × 10^7^ CFU in total), followed by bacterial load enumeration as described above. The competitive index (CI) was calculated by output (log_10_CFU1/log_10_CFU2)/Input (log_10_CFU1/log_10_CFU2). Δ*stp*, which was inframe substituted with the Erm^r^ coding sequence, has erythromycin resistance, which can be differentiated by plating on an erythromycin plate. The numbers of the Δ*stk* and Δ*stp*Δ*stk* strains in the recovered colonies were determined by using PCR.

### 2.6. Growth Assay

The growth assay was measured in a Bioscreen C system (Lab Systems Helsinki, Vantaa, Finland). To detect bacterial growth in TSB and CDM, overnight grown cell culture was diluted in TSB and CDM medium in a Bioscreen plate to an initial OD_600 nm_ of 0.1. The OD_600 nm_ was automatically monitored every 30 min at 37 °C with shaking. The assay was performed in triplicate.

### 2.7. Morphological Analysis

Light microscopy*,* scanning electron microscopy (SEM), and dual-color structured illumination microscopy (SIM) were used to analyze bacterial morphology. Bacteria were cultured in TSB containing 10% inactivated NBS overnight at 37 °C, diluted 1: 100 into fresh medium, grown to the mid-log phase, collected, and washed three times with PBS, stained with Gram staining reagents according to established procedures, and observed under a light microscope. Scanning electron microscopy (SEM) was performed as previously described [53], with some modifications. Bacteria at the mid-log phase were washed three times with PBS, spotted onto glass coverslips, fixed with 2.5% glutaraldehyde overnight at 4 °C, dehydrated with increasing concentrations of ethanol of 30% (15 min), 50% (15 min), 70% (15 min), 90% (15 min), and 100% (15 min, twice), air-dried, covered with a 10 nm gold/platinum layer (JSM-6390LV, JEOL, Japan), and observed with SEM (JFC-1600, JEOL, Tokyo, Japan). FDL, which is a kind of fluorescent D-amino acid dye (FDAA) [54], was used to label the newly synthesized peptidoglycan. Alexa Fluor™ 647 NHS Ester (AF-647) (Thermo Scientific, Waltham, MA, USA) was used to label the outline of *S. suis*. Bacteria were washed three times with PBS, mixed with FDL at a final concentration of 200 μM, incubated at 37 °C for 10 min, and washed three times with PBS. The resuspension was mixed with AF-647 at a final concentration of 20 nM, incubated at 28 °C for 30 min, washed three times with PBS, and imaged with dual-color structured illumination microscopy (SIM) (Nikon Instruments, Inc., Tokyo, Japan) (FDL, excitation at 490 nm and emission at 525 nm; AF-647, excitation at 651 nm and emission at 672 nm).

### 2.8. Trypsin Digestion

Triplicate cultures were harvested at the mid-log phase, resuspended in lysis buffer, and ultrasonicated (PTM Bio, Zhejiang, China). The protein was precipitated with precooled 20% trichloroacetic acid (TCA) for 2 h at −20 °C and redissolved in 8 M urea. The protein was digested overnight with trypsin (1: 50 trypsin: protein mass ratio).

### 2.9. TMT Labeling

The peptides were desalted using a Strata X C18 SPE column (Phenomenex), vacuum-dried, and reconstituted with 0.5 M TEAB according to the manufacturer’s protocol (Thermo Fisher Scientific, Waltham, MA, USA). Each sample was labeled with different tags, incubated for 2 h at room temperature, pooled, desalted, and dried by vacuum centrifugation.

### 2.10. HPLC Fractionation and Enrichment of Phosphorylated Peptides

Peptide fractions were acquired on a Thermo Betasil C18 column (5 μm particles, 10 mm ID, 250 mm length) through high pH reversed-phase HPLC. For enrichment of phosphomodified peptides, tryptic peptide mixtures were mixed with IMAC microspheres in loading buffer with gentle vibration. After centrifugation, the supernatant was removed, and IMAC microspheres with bound phosphopeptides were acquired. These IMAC microspheres were washed with 50% acetonitrile/6% trifluoroacetic acid and 30% acetonitrile/0.1% trifluoroacetic acid continuously to remove nonspecifically adsorbed peptides. To elute the enriched phosphopeptides from the IMAC microspheres, elution buffer containing 10% NH_4_OH was added, and the enriched phosphopeptides were eluted with vibration. Finally, the peptides were lyophilized for LC-MS/MS analysis.

### 2.11. LC-MS/MS Analysis

After the peptides were dissolved using 0.1% formic acid and separated with an EASY-nLC 1000 ultrahigh-performance liquid phase system, they were subjected to the NSI source followed by Orbitrap Fusion^TM^ system analysis. The NCE (set to 28) and Orbitrap (resolution = 17,500) were used to select peptides for MS/MS and detect the fragments.

### 2.12. Data Analysis

In this study, both proteomics and phosphoproteomics assays were conducted on the same cohort with the same batch of samples. The relative quantitative value of the phosphopeptide is divided by the relative quantitative value of corresponding proteins to remove the influence from protein expression. The MaxQuant search engine (v.1.5.2.8) was used to analyze the MS/MS data. Parameter setting: UniProt_Streptococcus_suis_strain_SC84 (2104 sequences). Image Pro Plus 6.0 was used for chain-length measurement. ImageJ was used to measure cell length and width.

### 2.13. Statistical Analysis

GraphPad Prism (version 8) software was used for statistical analysis. The Student’s *t*-test was used to analyze the differences between the two groups. Survival rates between different groups in the animal infection assay were analyzed by the log-rank (Mantel-Cox) test [55].

## 3. Results

### 3.1. Construction of S. suis Δstp, Δstk, and ΔstpΔstk Mutants

**Figure 1:**
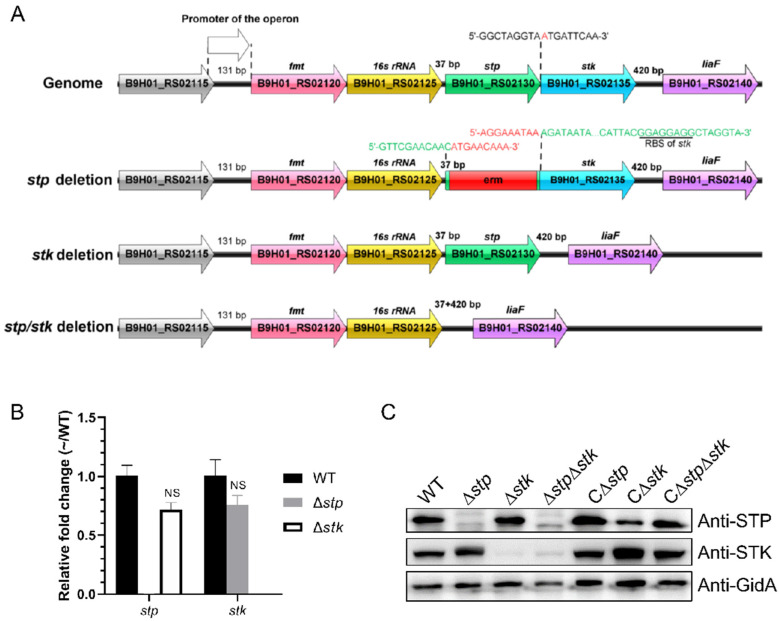
Construction and verification of Δ*stp*, Δ*stk*, and Δ*stp*Δ*stk.* (**A**) Schematic representation of the genome organization of the *stp*/*stk* operon in wild-type and mutant strains of *S. suis*. (**B**) Transcription levels of the *stp* and *stk* genes in the WT, ∆*stp,* and ∆*stk* strains. Total RNA was extracted from the WT, ∆*stp*, and ∆*stk* strains, respectively, and reverse-transcribed to cDNA, which was used as the template for RT-qPCR. The *gapdh* gene was used as the internal reference for normalization. Data are representative of three independent experiments with the mean ± standard deviation, and fold changes were calculated relative to wild-type expression levels. Fold change ≥ 1.5 and *p* value ≤ 0.05 were considered to represent a differential expression. (**C**) Detection of STP and STK expression by Western blot. Cells of each indicated strain at the mid-log phase were collected, washed with PBS, and lysed by sonication, followed by immunoblot analysis using STP, STK, and GidA polyclonal antibodies. Protein samples were normalized before loading. The GidA antibody was used as a control. C∆*stp*, C∆*stk*, and C∆*stp*∆*stk* indicate the corresponding complemented strains.

To investigate the functions of protein phosphorylation and dephosphorylation mediated by STK and STP in *S. suis*, we constructed three mutant strains, ∆*stp*, ∆*stk*, and ∆*stp*∆*stk* of *S. suis*, as well as their complement strains C∆*stp*, C∆*stk*, and C∆*stp*∆*stk*. As previously reported that *stp* and *stk* were cotranscribed [20], it was critical to ensure that deletion of *stp* did not affect the expression of *stk*. Therefore, the coding sequence (CDS) of the erythromycin resistance gene was used to replace the CDS of *stp* to avoid downstream polar effects (Figure 1A), and the expression of *stp* and *stk* in each strain was analyzed by RT-qPCR and Western blot. Figure 1B,C show that the expression level of STK was comparable in the ∆*stp* and WT strains at both the mRNA and protein levels. The complemented strains also showed restored expression of STP and STK, respectively. These results suggested that the mutant strains and their complement strains were constructed successfully.

### 3.2. The Role of STP, STK, and STP/STK in Bacterial Pathogenicity in Mice

**Figure 2:**
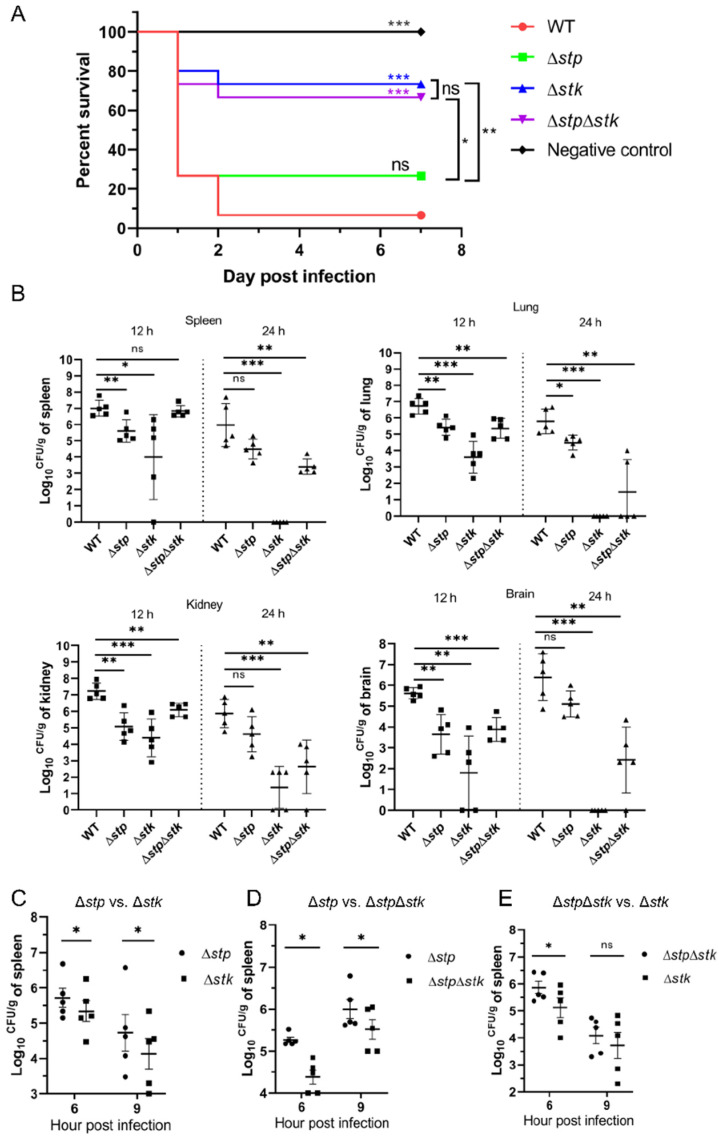
Virulence evaluation of the Δ*stp*, Δ*stk*, and Δ*stp*Δ*stk* strains. (**A**) Mouse survival assay. Mice were intraperitoneally injected with 8 × 10^8^ CFU of WT, Δ*stp*, Δ*stk*, or Δ*stp*Δ*stk*, respectively, and PBS was used as a negative control. Significant differences in survival rates between different groups were analyzed by the log-rank (Mantel-Cox) test. Fifteen mice were used in each group. (**B**) Bacterial colonization assay. Mice were intraperitoneally injected with 9 × 10^7^ CFU of WT, Δ*stp*, Δ*stk*, or Δ*stp*Δ*stk*, respectively. Mice were euthanized at each indicated time point. The spleen, lung, kidney, and brain were collected, resuspended in PBS, homogenized, and plated on TSA plates for colony enumeration. Five mice were used in each group. Data are presented as the mean ± standard deviations. Statistical significance is determined by two-tailed, unpaired Student’s *t*-tests (ns, *p* value > 0.05; *, *p* value < 0.05; **, *p* value < 0.01; ***, *p* value < 0.001). (**C**–**E**) Competitive infection assay. Mice were infected with a 1:1 mixture of Δ*stp* and Δ*stk* (**C**), Δ*stp* and Δ*stp*Δ*stk* (**D**), or Δ*stp*Δ*stk* and Δ*stk* (**E**). Spleens were collected, resuspended in PBS, homogenized, and plated on TSA plates for colony enumeration. Five mice were used in each group. Statistical significance was determined by two-tailed, unpaired Student’s *t*-tests (ns, *p* value > 0.05; *, *p* value < 0.05).

STP and STK are believed to be cognate pairs responsible for reversible protein phosphorylation, and they have both been shown to be involved in bacterial pathogenesis [34,35,56]. However, the exact role of each protein in *S. suis* pathogenesis remains to be further investigated. Therefore, we used a mouse infection model to compare the virulence of Δ*stp*, Δ*stk*, and Δ*stp*Δ*stk* strains with that of the WT strain of *S. suis*. In the mouse survival assay, only 7% (1/15) of the mice intraperitoneally injected with 8 × 10^8^ CFU of the WT strain survived. In comparison, the survival rates for the Δ*stp*, Δ*stk*, and Δ*stp*Δ*stk* strains were 27% (4/15), 73% (11/15), and 67% (10/15), respectively, indicating that *stk* played a more important role than *stp* in regulating the virulence of *S. suis* (Figure 2A). We further performed an in vivo colonization assay in mice with the WT strain and three mutants. The results showed that from 12 h post-infection (hpi) to 24 hpi, the bacterial load of the WT strain in each organ slowly decreased, indicating a strong ability to colonize the host. In contrast, the bacterial loads of the mutant strains showed significantly faster clearance in each organ. In particular, at 24 hpi, the Δ*stk* strain was almost completely cleared in the lung, spleen, and brain (Figure 2B), which was consistent with the results of the mouse survival assay. In contrast, the Δ*stp* and Δ*stp*Δ*stk* strains showed a significantly higher ability to colonize mice than the Δ*stk* strain. To more accurately compare the virulence of the mutants, we performed a series of competitive infection assays. As shown in Table 1 and Figure 2C–E, Δ*stp* significantly outcompeted Δ*stk* (CI_6h_ = 1.16746, *p* < 0.05; CI_9h_ = 1.24600, *p* < 0.05) and Δ*stp*Δ*stk* (CI_6h_ = 1.54531, *p* < 0.05; CI_9h_ = 1.39062, *p* < 0.05). Δ*stp*Δ*stk* showed a significantly higher bacterial load than Δ*stk* at 6 hpi (CI_6h_ = 1.26691, *p* < 0.05), but no significant difference in bacterial load was observed at 9 hpi (CI_9h_ = 1.24696, *p* > 0.05). Collectively, our data demonstrated that the deletion of *stk* significantly attenuated the virulence of *S. suis*, which was consistent with the observations in our previous reports [17]. In comparison, *stp* deletion resulted in reduced impact compared to the *stk* deletion on impairing the virulence of *S. suis*.

### 3.3. The Role of STP and STK in Growth and Cell Division

**Figure 3:**
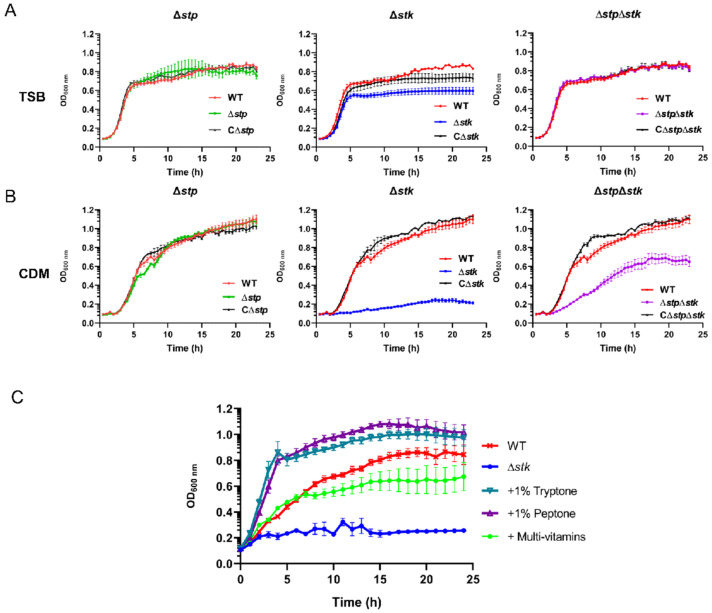
**Growth analysis.** Cells of the WT strain and each mutant grown overnight were inoculated in TSB medium (**A**) or CDM (**B**) with a similar initial OD_600 nm_ value. (**C**) The growth of WT strain in CDM and that of the Δ*stk* strain in CDM or CDM supplemented with each indicated nutrient. Growth was monitored with an automatic plate reader with shaking at 37 °C. Data are presented as means ± SD of triplicate.

We further tested other important phenotypes, including growth and cell division of the Δ*stp*, Δ*stk*, and Δ*stp*Δ*stk* strains. The growth of each mutant and WT strain in TSB medium or chemically defined minimal medium (CDM) was tested. As shown in Figure 3A, the growth of WT, Δ*stp*, and Δ*stp*Δ*stk* was comparable in TSB, whereas Δ*stk* presented retarded growth and reached a lower OD_600 nm_ in the stationary phase (Figure 3A). Surprisingly, the Δ*stk* strain could barely grow in CDM (Figure 3B). However, the growth of Δ*stp* was similar to that of the WT strain, and the growth of Δ*stp*Δ*stk* was much slower than that of WT but better than that of Δ*stk* (Figure 3B). To determine the cause of such growth defects, CDM supplemented with different carbon sources, casamino acids (CAAs), vitamins, purines, or complex nutrients (tryptone or peptone) was used to test the growth of the Δ*stk* strain (Figure 3C and Figure S1). As shown in Figure 3C, in addition to the observation that the growth of Δ*stk* was greatly increased in CDM supplemented with tryptone or peptone, it was interesting to see that its growth in CDM supplemented with multivitamins was also significantly recovered. These results suggested that STK was more important than STP for the growth of *S. suis*.

**Figure 4:**
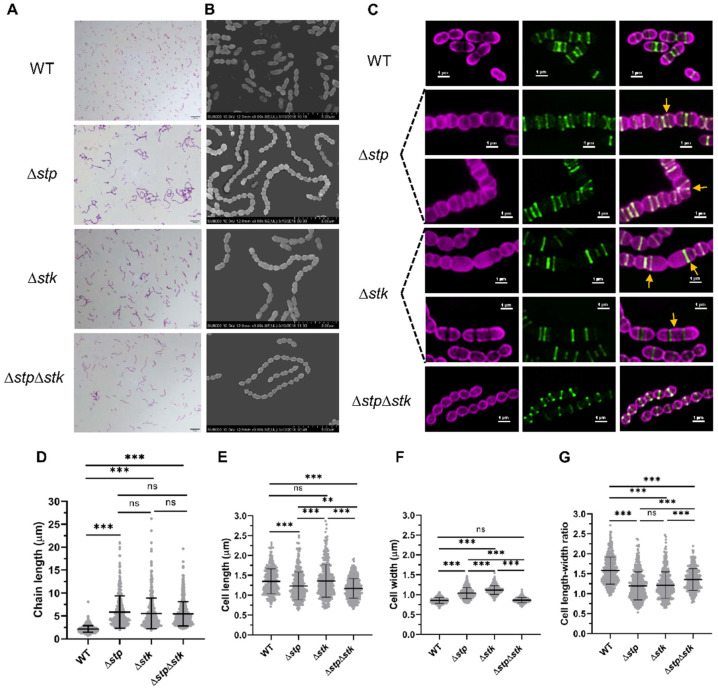
Morphological analysis of the Δ*stp*, Δ*stk*, and Δ*stp*Δ*stk* strains. (**A**) Gram staining images of the mid-log phase cells of WT, ∆*stp*, ∆*stk,* and ∆*stp*∆*stk* strains. The scale bars are 10 µm. (**B**) Scanning electron microscope analysis of the WT, ∆*stp*, ∆*stk,* and ∆*stp*∆*stk* strains. The scale bars are 5 µm. (**C**) Structured illumination microscopy (SIM) analysis of WT, ∆*stp*, ∆*stk,* and ∆*stp*∆*stk* strains. The mid-log phase cells were stained with AF-647 and FDL dyes, followed by imaging with SIM. The scale bars are 1 µm. (**D**) Chain length of WT, ∆*stp*, ∆*stk*, ∆*stp*∆*stk* strains. The results were obtained by measuring at least 400 chains per sample. (**E**–**G**) Cell size parameters of WT, ∆*stp*, ∆*stk*, and ∆*stp*∆*stk* strains. n_WT_ = 370, *n*_∆*stp*_ = 381, *n*_∆*stk*_ = 385, *n*_∆*stp*∆*stk*_ = 447. Data are presented as the mean ± standard deviations. Statistical significance was determined by two-tailed, unpaired Student’s *t*-tests (ns, *p* value > 0.05; **, *p* value < 0.01; ***, *p* value < 0.001).

STK has been reported to play important roles in cell division in *Streptococcus* [23,29], while limited information about the role of STP in cell division has been reported. Therefore, we subsequently analyzed the morphology, cell division, and nascent peptidoglycan synthesis of WT and mutant strains using light microscopy (LM), scanning electron microscopy (SEM), and dual-color structured illumination microscopy (SIM). One of the most significant morphological changes among the mutants was the increase in the length of bacterial chains. All three mutants formed long chains, with each chain comprising over ten cells compared with the WT strain, which showed a normal chain length with each chain harboring three to five cells (Figure 4A–D). The morphology of all the complemented strains was restored (Figure S2). These results revealed the presence of defects in cell division or daughter cell separation. Further analysis with SIM revealed abnormal cell division in both Δ*stp* and Δ*stk* strains. By staining with Alexa Fluor 647 dye (AF-647), which specifically labels the cytoplasmic membrane, and fluorescein-D-lysine (FDL), which labels nascent peptidoglycan (PG), followed by visualization using SIM, the cell morphology and division septum could be clearly observed (Figure 4C). Compared with the WT cells that displayed a standard ellipsoidal shape, 37.3% (142/381) of Δ*stp* cells showed a rounded morphology due to a decreased length-width ratio (Figure 4E–G). Moreover, in 9.4% (36/381) of Δ*stp* cells, the Z-ring was not localized to the mid-cell, and the angles of the Z-rings with respect to the cell long axis were incorrect, leading to the formation of twisted cell chains (Figure 4C, marked by yellow arrows). The Δ*stk* cells also showed abnormal cell division; however, they displayed a distinctly aberrant morphology. Approximately 6.8% (26/385) of the Δ*stk* cells underwent disordered division, leading to the formation of asymmetric daughter cells, and 10.4% (40/385) formed elongated cells containing multiple, unconstricted cell division rings (Figure 4C, marked with yellow arrows). In contrast, the Δ*stp*Δ*stk* strain showed relatively normal cell division. These results demonstrated that STP and STK both played an essential role in cell division.

### 3.4. Comparative Proteomic Analysis Revealing the Influence of stp and stk on Protein Expression

**Figure 5:**
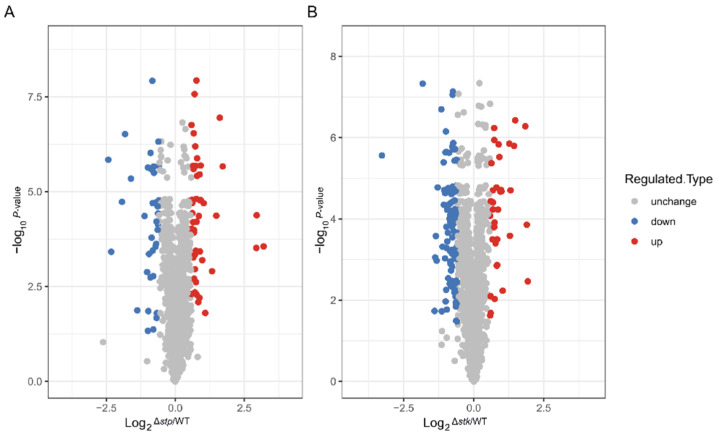
Comparative proteomic analysis between the WT and Δ*stp* or Δ*stk* strains. Volcano plots showing the differentially expressed proteins in ∆*stp* (**A**) or ∆*stk* (**B**) compared to WT strain. Fold change > 1.5 and *p* < 0.05 is regarded as significant change. Blue dots represent down-regulated proteins, red dots represent up-regulated proteins, and grey dots represent proteins with no significant change.

To investigate the influence of STP and STK on protein expression in *S. suis*, we performed a comparative proteomics analysis with the WT, Δ*stp*, and Δ*stk* strains using liquid chromatography mass spectrometry/mass spectrometry (LC-MS/MS), as illustrated in Figure S3A. A total of 1427 proteins were identified, constituting 72% of the proteins encoded by the *S. suis* SC19 genome. Among these identified proteins, the abundance of 1379 proteins was quantitatively compared between the WT and each mutant strain (Figure S3B). As shown in Figure 5A,B, compared with the WT strain, the Δ*stk* strain possessed the most dramatic change in protein abundance, in which 139 genes were differentially expressed, with 37 proteins upregulated and 102 proteins downregulated. For the Δ*stp* strain, 82 genes were differentially expressed compared with the WT strain, including 47 upregulated and 35 downregulated genes (fold change > 1.5, *p* < 0.05).

**Figure 6:**
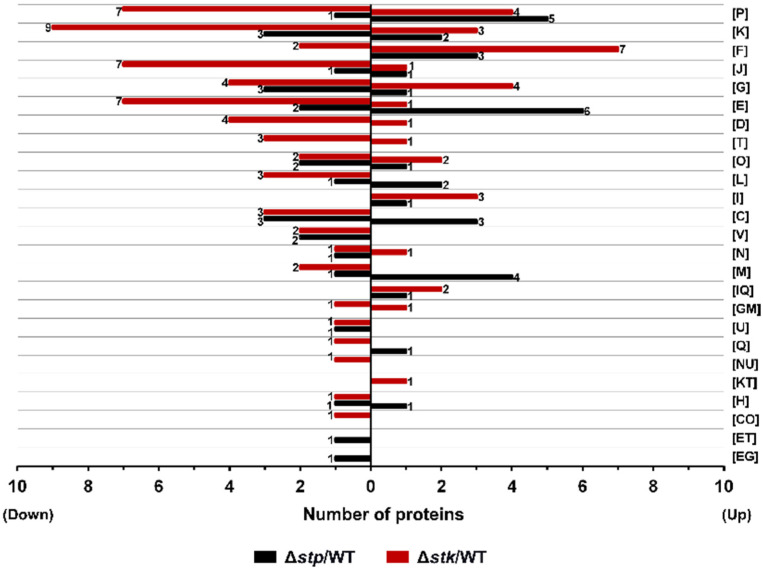
COG enrichment of the differentially expressed proteins in Δ*stp* and Δ*stk* strains. The differentially expressed proteins (fold change > 1.5, *p* value < 0.05) were analyzed with the EggNOG v5.0 database (http://eggnog5.embl.de/#/app/home (accessed on 25 June 2021)). [P] Inorganic ion transport and metabolism; [K] Transcription; [F] Nucleotide transport and metabolism; [J] Translation, ribosomal structure and biogenesis; [G] Carbohydrate transport and metabolism; [E] Amino acid transport and metabolism; [D] Cell cycle control, cell division, chromosome partitioning; [T] Signal transduction mechanisms; [O] Posttranslational modification, protein turnover, and chaperones; [L] Replication, recombination and repair; [I] Lipid transport and metabolism; [C] Energy production and conversion; [V] Defense mechanisms; [N] Cell motility; [M] Cell wall/membrane/envelope biogenesis; [IQ] Lipid transport and metabolism, Secondary metabolites biosynthesis, transport, and catabolism; [GM] Carbohydrate transport and metabolism, Cell wall/membrane/envelope biogenesis; [U] Intracellular trafficking, secretion, and vesicular transport; [Q] Secondary metabolites biosynthesis, transport, and catabolism; [NU] Cell motility, Intracellular trafficking, secretion, and vesicular transport; [KT] Transcription, Signal transduction mechanisms; [H] Coenzyme transport and metabolism; [CO] Energy production and conversion, Posttranslational modification, protein turnover, and chaperones; [ET] Amino acid transport and metabolism, Signal transduction mechanisms; [EG] Amino acid transport and metabolism, Carbohydrate transport and metabolism.

The EggNOG v5.0 database was used to analyze the functions of the differentially expressed proteins [57]. As shown in Figure 6, apart from the proteins with unknown functions, the differentially expressed proteins between the WT strain and each mutant were found to be distributed in almost all functional groups. Δ*stk* harbored the most downregulated proteins, in which the differentially expressed proteins most frequently fell in categories including K (Transcription), P (Inorganic ion transport and metabolism), J (Translation, ribosomal structure, and biogenesis), and G (Carbohydrate transport and metabolism). In contrast, Δ*stp* presented fewer downregulated proteins but had more proteins with increased abundance in which the differentially expressed proteins most frequently fell in categories including E (Amino acid transport and metabolism), P (Inorganic ion transport and metabolism), K (Transcription), and C (Energy production and conversion) (Figure 6). The data suggested that both STP and STK were global regulators of protein expression. As Δ*stk* showed retarded growth in CDM but the growth was restored in CDM supplemented with multivitamins, by analyzing the differentially expressed genes in the mutants, we found that a coA-binding protein (NZ_CP020863.1_prot_WP_012027318.1_1268) was significantly downregulated in Δ*stk* but was not differentially expressed in Δ*stp* (Table S3).

**Figure 7:**
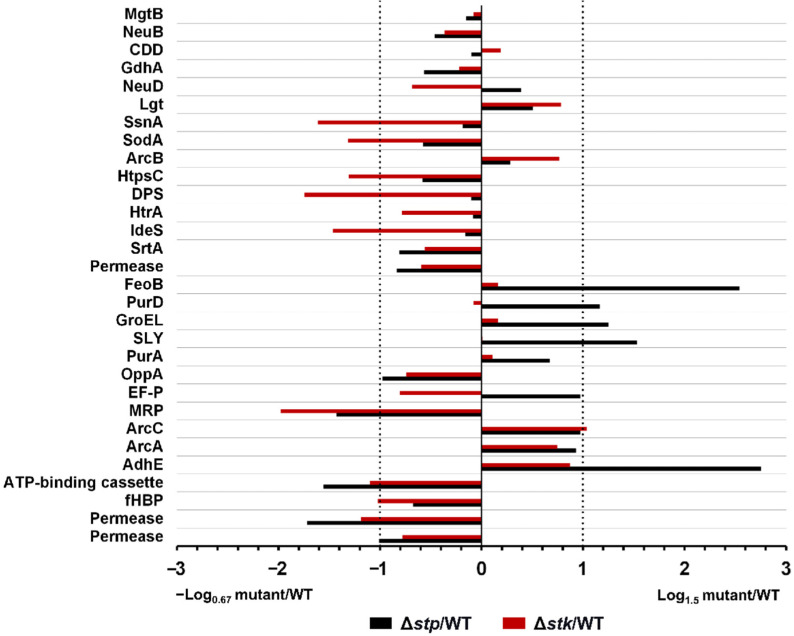
Expression of the virulence factors in Δ*stp* and Δ*stk* strains. The abundance of each indicated virulence factor in Δ*stp* or Δ*stk* strain was compared with that in WT, respectively. The proteins listed on the Y-axis are virulence factors of *S. suis*, and the numbers on the X-axis indicate the relative expression level compared with the WT strain. Fold change > 1.5 and *p* < 0.05 was regarded as significantly different.

As there was a difference in virulence between Δ*stp* and Δ*stk* strains, the abundance of several known virulence factors (VFs) in the mutant strains was compared with that in the WT strain [58]. As shown in Figure 7, among the 30 VFs, nine were significantly downregulated, including SsnA (B9H01_09460), SodA (B9H01_07435), HtpsC (B9H01_07610), DPS (B9H01_08150), IdeS (B9H01_02750), MRP (B9H01_03725), ATP-binding cassette (B9H01_06780), fHBP (B9H01_01430), and Permease (B9H01_06785), and only one, ArcC (B9H01_03140), was upregulated in Δ*stk*. In contrast, in Δ*stp*, only three VFs were significantly downregulated, including MRP (B9H01_03725), ATP-binding cassette (B9H01_06780), and Permease (B9H01_06785), and five VFs were upregulated, including FeoB (B9H01_06805), PurD (B9H01_00260), GroEL (B9H01_00900), SLY (B9H01_06770), and AdhE (B9H01_01465), compared with those in the WT strain. These results corresponded with the virulence phenotype associated with the disruption of STK with significantly attenuated virulence of *S. suis*, while deletion of STP showed a gently reduced virulence.

### 3.5. Comparative Phosphoproteomic Analysis

**Figure 8:**
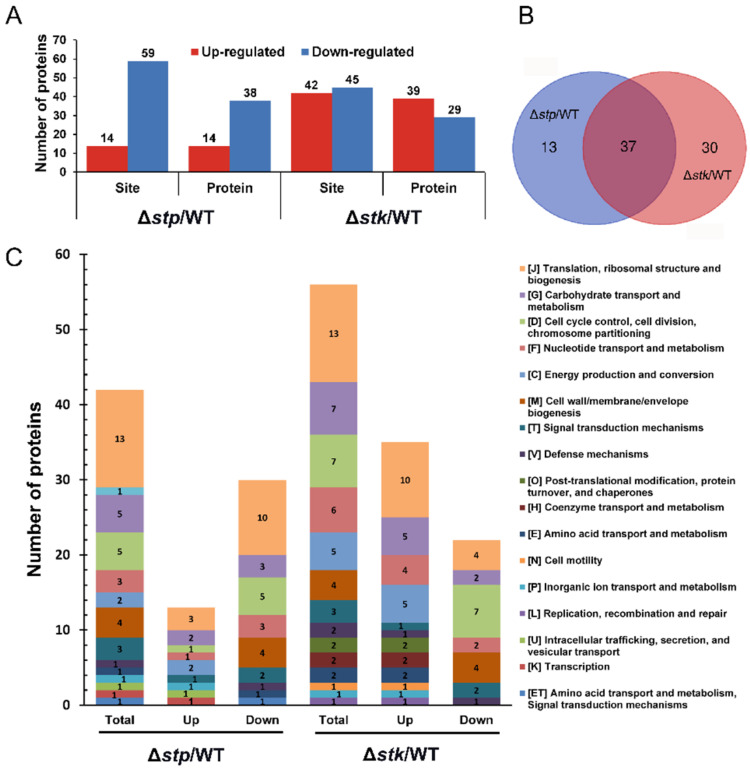
Comparative phosphoproteomic analysis between the WT and Δ*stp* or Δ*stk* strains. (**A**) Proteins identified with changed abundance after phosphopeptide enrichment between the WT and Δ*stp* or Δ*stk* strains. (**B**) Venn diagrams showing the proteins with changed phosphorylation levels in ∆*stp* and ∆*stk* compared with the WT strain. (**C**) COG enrichment of proteins with changed phosphorylation levels in the ∆*stp* and ∆*stk* compared with the WT strain. The COG enrichment analysis was performed using the EggNOG v5.0 database (http://eggnog5.embl.de/#/app/home (accessed on 25 June 2021)).

STK and STP are serine/threonine kinases and phosphatases, respectively. Therefore, we next analyzed the changes in the abundance of serine/threonine phosphorylated proteins in Δ*stp* and Δ*stk* compared with the WT strain. In Δ*stp*, a total of 73 unique phosphosites involving 50 proteins showed a significantly altered abundance (fold change > 1.5, *p* < 0.05), among which 14 proteins were less abundant and 38 proteins were more abundant compared with those in the WT strain. Between the Δ*stk* and WT strains, 87 unique phosphosites involving 67 proteins were identified with a changed abundance (fold change > 1.5, *p* < 0.05), among which 29 proteins were less abundant and 39 proteins were more abundant (Figure 8A and Table S4). A Venn diagram analysis showed that a total of 37 proteins were identified with changed abundance in both the Δ*stp* and Δ*stk* strains, suggesting potential overlapping functions of STP and STK (Figure 8B).

By COG analysis, it was shown that the phosphoproteins with changed abundance in both Δ*stp* and Δ*stk* strains were mainly related to “Translation, ribosomal structure and biogenesis”, “Carbohydrate transport and metabolism” and “Cell cycle control, cell division, chromosome partitioning”. In most of the COG categories, Δ*stk* presented more phosphoproteins with altered abundance than Δ*stp* (Figure 8C and Table S3), which coincided with the above observations that deletion of *stk* caused more severe phenotypic changes than *stp* deletion, including the impact on growth, metabolism, and virulence of *S. suis*.

**Figure 9:**
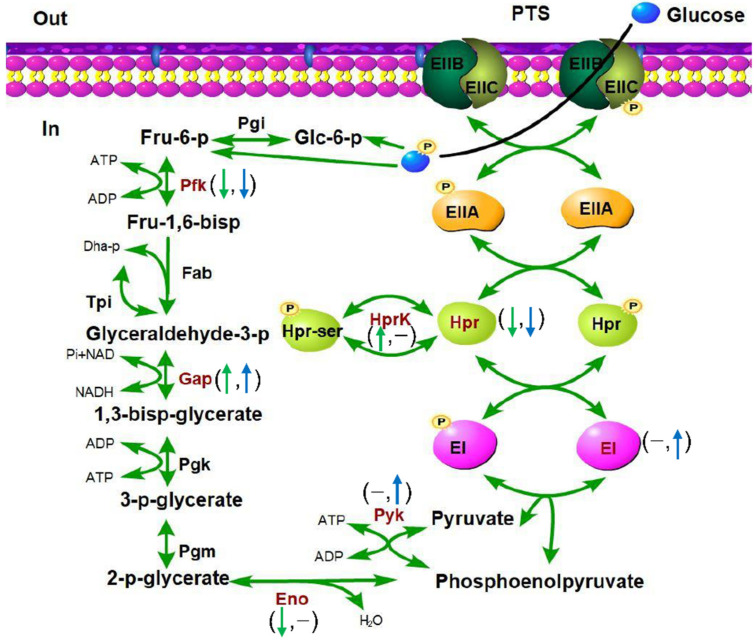
Proteins involved in bacterial glycolysis and carbohydrate transport with altered phosphorylation levels in Δ*stp* and Δ*stk*. The names of the proteins with altered phosphorylation levels are shown in dark red. In the brackets, the green arrow on the left indicates the change in phosphorylation level in the ∆*stp* strain compared with WT (*p* < 0.05), and the blue arrow on the right indicates the change in the ∆*stk* strain compared with WT (*p* < 0.05). The hyphen indicates no significant change (*p* > 0.05). Pgi, phosphoglucose isomerase; Pfk, phosphofructokinase; Fba, fructose-1,6-bisphosphate aldolase; Tpi, triosephosphate isomerase; Gap, glyceraldehyde-3-phosphate dehydrogenase; Pgk, phosphoglycerate kinase; Pgm, phosphoglycerate mutase; Eno, enolase; Pyk, pyruvate kinase; EI, PTS-enzyme I; Hpr, phosphocarrier protein; HprK, HPr kinase/phosphorylase.

Specifically, as shown in Table S4, several important proteins involved in central energy metabolism were identified to contain phosphosites with altered abundance in both Δ*stp* and Δ*stk* strains. HprK is a metabolic regulator that mediates the phosphorylation of HPr to control the metabolic rate [59]. The phosphorylation at S/T residues of Hpr was downregulated in Δ*stp* and Δ*stk*, while that of an S residue of HprK was upregulated only in Δ*stp*. The phosphorylation levels of GAPDH and GlmM were increased in both mutant strains compared with the WT strain, while the phosphorylation level of PfkA was decreased in both mutant strains compared with the WT strain. The phosphosite abundances of PtsEI and Pyk were increased in Δ*stk* and did not change in Δ*stp*. However, Eno exhibited an opposite situation (Figure 9).

Next, we analyzed the proteins with a reduced phosphorylation level in the Δ*stk* strain and increased phosphorylation level in the Δ*stp* strain, which might be their potential direct substrate proteins. Among the 29 proteins that showed a reduced abundance after enrichment of Ser/Thr phosphomodified peptides in Δ*stk*, a large proportion were cell division-related proteins, including MapZ, GpsB, MapZ, FtsZ, DivIVA, SepF, FtsW, MltG, Jag, and GlmS. The remaining proteins were mainly involved in metabolism, such as Hpr, NeuB, PfKA, ADK, GT, PhoH, and LmrC, and protein translation, such as InfB, EF-P, EF-G, and BipA (Table 2). In contrast, among the fourteen proteins that showed increased abundance after Ser/Thr phosphomodified peptide enrichment in the Δ*stp* strain, only four proteins, including DivIVA, STK, MltG, and GlmM, were involved in cell division, and the others were involved in metabolism, such as NADP, PrfA, GAPDH, OppF, Hprk, ArgS, SecA, and IDH (Table 2). These results suggested that STP and STK might affect the cell division, energy metabolism, and protein translation of *S. suis* in a manner dependent on Ser/Thr protein phosphorylation.

## 4. Discussion

The eukaryote-like Ser/Thr protein kinases (STKs) and phosphatases (STPs) in bacteria have recently attracted extensive research interest due to their important roles in cell division, metabolism, and pathogenesis in many important bacterial pathogens [22,35,56,60,61,62]. STKs and STPs comprise a complex regulatory system, of which the mechanism of regulation has not been fully understood. Many studies have been performed to reveal the functions of STKs and STPs, however, most of them focus on STK or STP individually. To provide a holistic understanding of this complex regulatory system, systemic investigations are needed. Therefore, we performed a comparative study in *S. suis*, an important zoonotic bacterial pathogen where only one copy of gene encoding STK and its cognate STP is present, with ∆*stk*, ∆*stp*, ∆*stk*∆*stp,* and the wild-type strains. Comprehensive phenotypic analysis combined with comparative proteomics and phosphoproteomics analysis were carried out to reveal the STK/STP mediated regulation network.

In the animal infection assay, we showed that STK was essential for the virulence of *S. suis*, which is consistent with previous studies [17,20]. Some previous studies also showed that deletion of *stp* led to decreased virulence [42,63]. Similarly, we presented detailed data showing that *stp* deletion also compromised the virulence of *S. suis*, however, the degree in virulence attenuation was much lower than that of *stk* deletion. The comparative proteomic results also revealed that more VFs showed downregulated expression in the Δ*stk* strain than in the Δ*stp* strain, which could potentially explain the difference in virulence between the two strains.

In the growth assay, it is interesting to see that the growth of the Δ*stk* strain was almost abolished in CDM, and the addition of multivitamins largely restored this growth defect. Among the differentially expressed proteins in Δ*stk*, we found that coA-binding protein was significantly downregulated (Table S3) and was not differentially expressed in Δ*stp*, which might explain the partial recovering effect of multivitamins on Δ*stk.*

It was noticed that in the results of both virulence evaluation and growth assays found that the double deletion mutant did not display a more severe attenuation than the single deletion mutant Δ*stk*. One possible explanation could be that potential phosphorylation systems in addition to the Ser/Thr kinase-phosphatase system may exist that have common targets. Indeed, crosstalk between different phosphorylation systems has been reported [64,65]. Under this circumstance, when STK and STP are both deleted, the substrate proteins may be phosphorylated by other phosphorylation systems, therefore giving phenotypes that are less severe than each of the single deletion mutants.

Bacterial cell division is a vital process requiring sophisticated spatial and temporal regulation to maintain accurate proliferation and proper morphology [66,67]. Our results showed that both Δ*stp* and Δ*stk* displayed a chained morphology, which was consistent with previous studies of that impaired in cell division [22,62] and peptidoglycan synthesis [23,33,68]. Comparative phosphoproteomic data in this study revealed that 31% of the proteins with a decreased phosphorylation level in the Δ*stk* strain were cell division related proteins, including MapZ, GpsB, FtsZ, DivIVA, SepF, FtsW, MltG [69], Jag [70], and GlmS [71]. Among these proteins, MapZ [26], GpsB [72], FtsZ [28], DivIVA [24], and Jag [73] have been demonstrated to be direct substrates of STK, while whether SepF, FtsW, MltG, and GlmS are STK substrates needs further confirmation. In the Δ*stp* strain, DivIVA, STK, MltG, and GlmM also showed increased phosphorylation levels, suggesting that these proteins may be common targets of STP and STK. The high prevalence of cell division-related proteins among the proteins with changed phosphorylation states in Δ*stp* and Δ*stk* further suggests the important roles of STP and STK in *S. suis* cell division.

Other than cell division-related proteins, it was observed that in Δ*stk,* several translation-related proteins also showed a downregulated phosphorylation level, which is consistent with previous studies [27,37]. Although other phosphatases also exist in *S. suis*, our phosphoproteomic data showed that the phosphorylation level of a variety of proteins was significantly changed in Δ*stp*, indicating an important role of STP. We also performed a phosphatase activity assay using the method previously described [74] with the WT, Δ*stp*, and CΔ*stp* strains, which showed that the total phosphatase activity of the Δ*stp* stain was indeed significantly lower than that of the WT strain (Figure S4). It is worth noting that Table 2 shows the proteins with downregulated phosphorylation in the Δ*stk* strain and upregulated phosphorylation in the Δ*stp* strain, which are more likely to be direct target proteins. As shown in Table S4, other proteins had upregulated phosphorylation levels in the Δ*stk* strain and downregulated phosphorylation levels in the Δ*stp* strain. The influence of STP and STK on the phosphorylation of these proteins could be an indirect effect. When comparing the proteins with altered phosphorylation in the Δ*stk* strain and Δ*stp* strain, there were some overlapping proteins, such as DivIVA and MltG. However, many more of the proteins were found specifically in each individual mutant strain. These data suggest that STP and STK functionally overlap but may also have specific functions, which has also been reported in previous studies. For example, in *S. pneumonia*, protein including Pgm, ComB, and RsMF are found to have changed phosphorylation status in Δ*stp* but not in Δ*stk* by the phosphoproteomics analysis, indicating the presence of potential STP-specific substrates [8]. In *S. pyogenes*, STP is shown to be secreted to the host cells to potentially activate proapoptotic signaling cascades; however, its cognate STK is unlikely to interfere with the host cell signaling [39]

In this study, comparative proteomics and phosphoproteomics analysis were used to reveal how STK and STP affect the growth, cell division, and virulence of *S. suis*. However, the omics data can just provide some clues. More experiments are needed to further reveal the underlying mechanisms. For example, questions, such as how STK affects the cell division of *S. suis* and which substrate proteins are responsible for the formation of the aberrant morphology and mislocalized Z-ing, and why STK deletion causes different growth defects in different growth media need further elucidation. Moreover, in the phosphoproteomics analysis, a variety of proteins showed downregulated phosphorylation in Δ*stk* and upregulated phosphorylation in Δ*stp*. These could be their potential substrate proteins. However, more studies are needed to verify them.

## 5. Conclusions

Although the STK and cognate STP are involved in the reversible protein phosphorylation, this study suggests that STK may play a much more important role in *S. suis* growth and pathogenesis than STP. Both ∆*stp* and ∆*stk* were defective in cell division, where they displayed an aberrant cell morphology. The ∆*stk* strain presented many more differentially expressed proteins than the ∆*stp* strain when compared with the wild-type strain. Many more known virulence factors were downregulated in ∆*stk* than in ∆*stp*. In ∆*stk* a substantial number of the proteins with reduced abundance after phosphopeptide enrichment were involved in cell division, followed by energy metabolism and protein translation. However, only a few proteins showed an increased abundance after phosphopeptide enrichment, which was also related to cell division. Collectively, both STP and STK are critical regulatory proteins in *S. suis*, and STK may play a more important role than STP in growth, cell division, and pathogenesis.

## Figures and Tables

**Table 1:** Competitive index (*n* = 5).

Strains	Mean CI		*p* Value		Significance	
	6 h	9 h	6 h	9 h	6 h	9 h
∆*stp* vs. ∆*stk*	1.16746	1.24600	0.01146	0.03982	*	*
∆*stp* vs. ∆*stp*∆*stk*	1.54531	1.39062	0.01170	0.02246	*	*
∆*stp*∆*stk* vs. ∆*stk*	1.26691	1.24696	0.01964	0.12846	*	NS

Note: *n* is the number of animals in each group. CI = Output (log_10_CFU1/log_10_CFU2)/Input (log_10_CFU1/log_10_CFU2). * indicates *p* value < 0.05; NS indicates no statistical significance.

**Table 2:** Proteins with down- and up-regulated abundance after phospho-peptide enrichment in Δ*stk* and Δ*stp* compared with WT.

No.	Protein Name	Protein Description	Amino Acid	Position	Regulated Type	Ratio	*p* Value	Function
Δ*stk*/WT								
1	GpsB	cell division regulator GpsB	S	73	Down	0.084	0.0008	Cell division
			T	86	Down	0.246	0.0298	
			T	66	Down	0.499	0.0257	
2	MapZ	Midcell-anchored protein Z	T	66	Down	0.075	4 × 10^−5^	
			T	26	Down	0.107	4 × 10^−6^	
3	FtsZ	cell division protein FtsZ	T	356	Down	0.483	0.0026	
4	DivIVA	DivIVA domain-containing protein	T	211	Down	0.289	0.0006	
			T	199	Down	0.46	8 × 10^−5^	
5	SepF	cell division protein SepF	S	179	Down	0.397	0.0033	
6	FtsW	FtsW/RodA/SpoVE family cell cycle protein	T	402	Down	0.128	4 × 10^−5^	
7	Jag	protein jag	T	87	Down	0.321	0.0002	
			T	129	Down	0.413	1 × 10^−6^	
			S	103	Down	0.555	1 × 10^−6^	
			S	84	Down	0.615	0.0385	
8	MltG	endolytic transglycosylase MltG	T	211	Down	0.107	0.0137	
			T	10	Down	0.109	0.0005	
			T	64	Down	0.147	0.0009	
			T	197	Down	0.203	8 × 10^−5^	
			T	122	Down	0.239	6× 10^−5^	
9	GlmS	glutamine-fructose-6-phosphate transaminase (isomerizing)	T	235	Down	0.12	8 × 10^−9^	
10	Hpr	phosphocarrier protein HPr	S	31	Down	0.621	4 × 10^−5^	Metabolism
			S	27	Down	0.624	0.0013	
			T	34	Down	0.665	0.0007	
11	NeuB	N-acetylneuraminate synthase	T	69	Down	0.339	0.006	
12	PfKA	ATP-dependent 6-phosphofructokinase	T	240	Down	0.596	0.0002	
13	ADK	adenylate kinase	T	137	Down	0.547	4 × 10^−5^	
14	GT	glycosyltransferase	T	441	Down	0.054	1 × 10^−7^	
15	-	phosphotransferase	T	5	Down	0.14	0.0013	
16	PhoH	phosphate starvation-inducible protein PhoH	T	328	Down	0.31	1 × 10^−4^	
17	LmrC	ABC transporter ATP-binding protein	T	324	Down	0.208	8 × 10^−8^	
18	InfB	translation initiation factor IF-2	T	326	Down	0.534	0.0043	Translation
19	EF-P	elongation factor P	T	144	Down	0.188	0.0162	
20	EF-G	elongation factor G	T	43	Down	0.312	2 × 10^−5^	
21	BipA	translational GTPase TypA	T	559	Down	0.26	0.0007	
22	-	PASTA domain-containing protein	T	25	Down	0.263	0.0024	Other
			S	30	Down	0.374	0.0071	
23	-	nucleoid-associated protein	T	244	Down	0.605	0.0016	
24	-	IreB family regulatory phosphoprotein	T	7	Down	0.35	2 × 10^−5^	
25	-	50S ribosomal protein L7/L12	T	16	Down	0.182	2 × 10^−5^	
26	-	Putative exported protein	T	72	Down	0.026	2 × 10^−7^	
		Putative exported protein	T	38	Down	0.433	4 × 10^−7^	
27	-	Putative membrane protein	T	4	Down	0.093	3 × 10^−6^	
28	-	Putative exported protein	T	80	Down	0.075	0.0392	
			T	114	Down	0.559	0.0079	
29	-	hypothetical protein	T	48	Down	0.044	8 × 10^−5^	
Δ*stp*/WT								
1	DivIVA	DivIVA domain-containing protein	T	199	Up	2.674	2 × 10^−5^	Cell division
2	STK	Stk1 family PASTA domain-containing Ser/Thr kinase	T	50	Up	7.981	0.0002	
3	MltG	endolytic transglycosylase MltG	S	115	Up	1.56	0.0141	
4	GlmM	phosphoglucosamine mutase	S	101	Up	1.525	0.0009	
5	NADP	NADP-dependent isocitrate dehydrogenase	S	102	Up	2.705	0.0034	Metabolism
6	PrfA	peptide chain release factor 1	S	297	Up	2.329	0.0042	
7	GAPDH	type I glyceraldehyde-3-phosphate dehydrogenase	T	212	Up	2.253	0.0004	
8	OppF	ABC transporter ATP-binding protein	S	298	Up	2.107	0.0013	
9	Hprk	HPr kinase/phosphorylase	S	300	Up	1.7	0.0008	
10	ArgS	arginine--tRNA ligase	S	187	Up	1.626	2 × 10^−5^	
11	SecA	preprotein translocase subunit SecA	S	808	Up	1.604	8 × 10^−5^	
12	IDH	L-lactate dehydrogenase	S	225	Up	1.516	8 × 10^−5^	
13	-	CsbD family protein	S	2	Up	1.895	0.0185	Other
14	-	30S ribosomal protein S3	S	168	Up	1.596	0.0249	

Note: The assay was performed with triplicate and the ratio was presented as the average protein abundance. The *p* value indicates the statistical difference of each protein between the mutant strain and the WT strain by using the Student’s *t*-test.

## Data Availability

The mass spectrometry proteomics data have been deposited to the ProteomeXchange Consortium via the PRIDE partner repository with the dataset identifier PXD029994.

## Supplementary Materials

The following are available online at https://www.mdpi.com/article/10.3390/microorganisms9122442/s1. Figure S1: Growth analysis of ∆stk strains. (A) The growth of ∆stk strain in CDM or CDM supplemented with each indicated nutrient. Growth was monitored with an automatic plate reader with shaking at 37 °C. Data are presented as means ± SD of triplicate. Figure S2: Morphological analysis of C∆stp, C∆stk, and C∆stp∆stk strains. Gram staining (A) and scanning electron microscopy (SEM) (B) analysis of C∆stp, C∆stk, and C∆stp∆stk strains. Figure S3: Comparative proteomic analysis between the WT and Δstp or Δstk strains. (A) The systematic workflow of the quantitative profiling of the global mass spectrum; (B) The basic statistics of mass spectrum data. Figure S4: Phosphatase activity assay. Cells at the mid-log phase of each indicated strain were collected, washed three times with buffer1 [20 mM Tris-HCl (pH 8.0) and 200 mM NaCl2], resuspended in lyse buffer [50 mM Tris-HCl (pH 8.0), 150 mM NaCl2, 100 U/mL mutanolysin (Sigma), and 1 mM PMSF], and lysed by sonication. The amount of total protein loaded was normalized using a Micro BCA protein assay kit (Cwbiotech, China). Samples were mixed with buffer2 [50 mM Tris-HCl (pH 8.0), 2 mM MnCl_2_, and 20 mM p-nitrophenyl phosphate (p-NPP)], and incubated at 37 °C for 10 min. The absorbance at 405 nm was then measured. Purified recombinant STP protein was used as the positive control, the reaction buffer2 used as the blank. Data are presented as means ± SD of triplicate. Statistical significance was determined by two-tailed, unpaired Student’s *t*-tests (ns, p value > 0.05; *, p value < 0.05). Table S1: Strains and plasmids used in this study. Table S2: oligonucleotides used in this study. Table S3: The differentially expressed proteins in ∆stp and ∆stk compared with WT. Table S4: Proteins identified with changed abundance after phosphopeptide enrichment in ∆*stp* and ∆*stk* compared with WT.

## References

[1] Hunter T. (1995). Protein Kinases and Phosphatases: The Yin and Yang of Protein Phosphorylation and Signaling. Cell.

[2] Goulian M. (2010). Two-Component Signaling Circuit Structure and Properties. Curr. Opin. Microbiol..

[3] Pereira S.F.F., Goss L., Dworkin J. (2011). Eukaryote-Like Serine/Threonine Kinases and Phosphatases in Bacteria. Microbiol. Mol. Biol. Rev..

[4] Shi L., Ji B., Kolar-Znika L., Boskovic A., Jadeau F., Combet C., Grangeasse C., Franjević D., Talla E., Mijakovic I. (2014). Evolution of Bacterial Protein-Tyrosine Kinases and Their Relaxed Specificity Toward Substrates. Genome Biol. Evol..

[5] Shi L., Pigeonneau N., Ravikumar V., Dobrinic P., Macek B., Franjević D., Noirot-Gros M.-F., Mijakovic I. (2014). Cross-Phosphorylation of Bacterial Serine/Threonine and Tyrosine Protein Kinases on Key Regulatory Residues. Front. Microbiol..

[6] Osaki M., Arcondéguy T., Bastide A., Touriol C., Prats H., Trombe M.-C. (2009). The StkP/PhpP Signaling Couple in *Streptococcus pneumoniae*: Cellular Organization and Physiological Characterization. J. Bacteriol..

[7] Nováková L., Sasková L., Pallová P., Janeček J., Novotná J., Ulrych A., Echenique J., Trombe M.-C., Branny P. (2005). Characterization of a Eukaryotic Type Serine/Threonine Protein Kinase and Protein Phosphatase of *Streptococcus pneumoniae* and Identification of Kinase Substrates. FEBS J..

[8] Hirschfeld C., Mejia A.G., Bartel J., Hentschker C., Rohde M., Maaß S., Hammerschmidt S., Becher D. (2019). Proteomic Investigation Uncovers Potential Targets and Target Sites of Pneumococcal Serine-Threonine Kinase StkP and Phosphatase PhpP. Front. Microbiol..

[9] Refaya A.K., Sharma D., Kumar V., Bisht D., Narayanan S. (2016). A Serine/threonine kinase PknL is involved in the Adaptive Response of *Mycobacterium tuberculosis*. Microbiol. Res..

[10] Malhotra V., Arteaga-Cortés L.T., Clay G., Clark-Curtiss J.E. (2010). *Mycobacterium tuberculosis* protein kinase K Confers Survival Advantage During Early Infection in Mice and Regulates Growth in Culture and during Persistent Infection: Implications for Immune Modulation. Microbiology.

[11] Débarbouillé M., Dramsi S., Dussurget O., Nahori M.-A., Vaganay E., Jouvion G., Cozzone A., Msadek T., Duclos B. (2009). Characterization of a Serine/Threonine Kinase Involved in Virulence of *Staphylococcus aureus*. J. Bacteriol..

[12] Liu Q., Fan J., Niu C., Wang D., Wang J., Wang X., Villaruz A.E., Li M., Otto M., Gao Q. (2011). The Eukaryotic-Type Serine/Threonine Protein Kinase Stk is Required for Biofilm Formation and Virulence in *Staphylococcus epidermidis*. PLoS ONE.

[13] Echenique J., Kadioglu A., Romao S., Andrew P.W., Trombe M.-C. (2004). Protein Serine/Threonine Kinase StkP Positively Controls Virulence and Competence in *Streptococcus pneumoniae*. Infect. Immun..

[14] Herbert J., Mitchell A., Mitchell T. (2015). A Serine-Threonine Kinase (StkP) Regulates Expression of the Pneumococcal Pilus and Modulates Bacterial Adherence to Human Epithelial and Endothelial Cells in Vitro. PLoS ONE.

[15] Bugrysheva J., Froehlich B.J., Freiberg J.A., Scott J.R. (2011). Serine/Threonine Protein Kinase Stk is Required for Virulence, Stress Response, and Penicillin Tolerance in *Streptococcus pyogenes*. Infect. Immun..

[16] Jin H., Pancholi V. (2006). Identification and Biochemical Characterization of a Eukaryotic-type Serine/Threonine Kinase and its Cognate Phosphatase in *Streptococcus pyogenes*: Their Biological Functions and Substrate Identification. J. Mol. Biol..

[17] Zhang C., Sun W., Tan M., Dong M., Liu W., Gao T., Li L., Xu Z., Zhou R. (2017). The Eukaryote-Like Serine/Threonine Kinase STK Regulates the Growth and Metabolism of Zoonotic *Streptococcus suis*. Front. Cell. Infect. Microbiol..

[18] Rui L., Weiyi L., Yu M., Hong Z., Jiao Y., Zhe M., Hongjie F. (2018). The Serine/Threonine Protein Kinase of *Streptococcus Suis* Serotype 2 Affects the Ability of the Pathogen to Penetrate the Blood-Brain Barrier. Cell Microbiol..

[19] Zhu H., Zhou J., Wang D., Yu Z., Li B., Ni Y., He K. (2021). Quantitative Proteomic Analysis reveals that Serine/Threonine Kinase is involved in *Streptococcus suis* Virulence and Adaption to Stress Conditions. Arch. Microbiol..

[20] Zhu H., Zhou J., Ni Y., Yu Z., Mao A., Hu Y., Wang W., Zhang X., Wen L., Li B. (2014). Contribution of Eukaryotic-Type Serine/Threonine Kinase to Stress Response and Virulence of *Streptococcus suis*. PLoS ONE.

[21] Liu H., Ye C., Fu H., Yue M., Li X., Fang W. (2021). Stk and Stp1 participate in *Streptococcus suis* serotype 2 Pathogenesis by Regulating Capsule Thickness and Translocation of Certain Virulence Factors. Microb. Pathog..

[22] Beilharz K., Nováková L., Fadda D., Branny P., Massidda O., Veening J.-W. (2012). Control of Cell Division in *Streptococcus pneumoniae* by the Conserved Ser/Thr Protein Kinase StkP. Proc. Natl. Acad. Sci. USA.

[23] Zucchini L., Mercy C., Garcia P.S., Cluzel C., Gueguen-Chaignon V., Galisson F., Freton C., Guiral S., Brochier-Armanet C., Gouet P. (2018). PASTA repeats of the Protein Kinase StkP Interconnect Cell Constriction and Separation of *Streptococcus pneumoniae*. Nat. Microbiol..

[24] Ni H., Fan W., Li C., Wu Q., Hou H., Hu D., Zheng F., Zhu X., Wang C., Cao X. (2018). *Streptococcus suis* DivIVA Protein Is a Substrate of Ser/Thr Kinase STK and Involved in Cell Division Regulation. Front. Cell. Infect. Microbiol..

[25] Kang C.M., Nyayapathy S., Lee J.Y., Suh J.W., Husson R.N. (2008). Wag31, a Homologue of the Cell Division Protein DivIVA, Regulates Growth, Morphology and Polar Cell Wall Synthesis in Mycobacteria. Microbiology.

[26] Fleurie A., Lesterlin C., Manuse S., Zhao C., Cluzel C., Lavergne J.-P., Franz-Wachtel M., Macek B., Combet C., Kuru E. (2014). MapZ Marks the Division Sites and Positions FtsZ rings in *Streptococcus pneumoniae*. Nature.

[27] Pompeo F., Foulquier E., Serrano B., Grangeasse C., Galinier A. (2015). Phosphorylation of the Cell Division Protein GpsB regulates PrkC Kinase Activity through a Negative Feedback Loop in *Bacillus subtilis*. Mol. Microbiol..

[28] Maurya G.K., Modi K., Banerjee M., Chaudhary R., Rajpurohit Y.S., Misra H.S. (2018). Phosphorylation of FtsZ and FtsA by a DNA Damage-Responsive Ser/Thr Protein Kinase Affects their Functional Interactions in *Deinococcus radiodurans*. mSphere.

[29] Fenton A.K., Manuse S., Flores-Kim J., Garcia P.S., Mercy C., Grangeasse C., Bernhardt T.G., Rudner D.Z. (2018). Phosphorylation-Dependent Activation of the Cell Wall Synthase PBP2a in *Streptococcus Pneumoniae* by MacP. Proc. Natl. Acad. Sci. USA.

[30] Li W., Yin Y., Meng Y., Ma Z., Lin H., Fan H. (2021). The Phosphorylation of Phosphoglucosamine Mutase GlmM by Ser/Thr Kinase STK Mediates Cell Wall Synthesis and Virulence in *Streptococcus suis* serotype 2. Veter. Microbiol..

[31] Falk S.P., Weisblum B. (2013). Phosphorylation of the *Streptococcus pneumoniae* Cell Wall Biosynthesis Enzyme MurC by a Eukaryotic-Like Ser/Thr kinase. FEMS Microbiol. Lett..

[32] Iswahyudi, Mukamolova G.V., Straatman-Iwanowska A.A., Allcock N., Ajuh P., Turapov O., O’Hare H.M. (2019). Mycobacterial phosphatase PstP regulates Global Serine Threonine Phosphorylation and Cell Division. Sci. Rep..

[33] Jarick M., Bertsche U., Stahl M., Schultz D., Methling K., Lalk M., Stigloher C., Steger M., Schlosser A., Ohlsen K. (2018). The Serine/Threonine Kinase Stk and the Phosphatase Stp Regulate Cell Wall Synthesis in *Staphylococcus aureus*. Sci. Rep..

[34] Sharma A.K., Arora D., Singh L.K., Gangwal A., Sajid A., Molle V., Singh Y., Nandicoori V.K. (2016). Serine/Threonine Protein Phosphatase PstP of *Mycobacterium tuberculosis* is Necessary for Accurate Cell Division and Survival of Pathogen. J. Biol. Chem..

[35] Sajid A., Arora G., Singhal A., Kalia V.C., Singh Y. (2015). Protein Phosphatases of Pathogenic Bacteria: Role in Physiology and Virulence. Annu. Rev. Microbiol..

[36] Agarwal S., Pancholi P., Pancholi V. (2012). Strain-Specific Regulatory Role of Eukaryote-Like Serine/Threonine Phosphatase in Pneumococcal Adherence. Infect. Immun..

[37] Wright D.P., Ulijasz A.T. (2014). Regulation of Transcription by Eukaryotic-Like Serine-Threonine Kinases and Phosphatases in Gram-Positive Bacterial Pathogens. Virulence.

[38] Schultz C., Niebisch A., Schwaiger A., Viets U., Metzger S., Bramkamp M., Bott M. (2009). Genetic and Biochemical Analysis of the Serine/Threonine Protein Kinases PknA, PknB, PknG and PknL of *Corynebacterium glutamicum*: Evidence for Non-Essentiality and for Phosphorylation of OdhI and FtsZ by Multiple Kinases. Mol. Microbiol..

[39] Agarwal S., Agarwal S., Jin H., Pancholi P., Pancholi V. (2012). Serine/Threonine Phosphatase (SP-STP), Cecreted from *Streptococcus pyogenes*, Is a Pro-apoptotic Protein. J. Biol. Chem..

[40] Segura M., Calzas C., Grenier D., Gottschalk M. (2016). Initial Steps of the Pathogenesis of the Infection caused by *Streptococcus suis*: Fighting against Nonspecific Defenses. FEBS Lett..

[41] Zhu H., Huang D., Zhang W., Wu Z., Lu Y., Jia H., Wang M., Lu C. (2011). The Novel Virulence-Related Gene *stp* of *Streptococcus suis* serotype 9 strain Contributes to a Significant Reduction in Mouse Mortality. Microb. Pathog..

[42] Fang L., Zhou J., Fan P., Yang Y., Shen H., Fang W. (2017). A Serine/Threonine Phosphatase 1 of *Streptococcus suis* type 2 is an Important Virulence Factor. J. Veter. Sci..

[43] Li W., Liu L., Chen H., Zhou R. (2009). Identification of *Streptococcus suis* Genes Preferentially Expressed under Iron Starvation by Selective Capture of Transcribed Sequences. FEMS Microbiol. Lett..

[44] van de Rijn I., Kessler R.E. (1980). Growth Characteristics of Group A Streptococci in a New Chemically Defined Medium. Infect. Immun..

[45] Trieu-Cuot P., Carlier C., Poyart-Salmeron C., Courvalin P. (1991). Shuttle Vectors Containing a Multiple Cloning Site and a *lacZ* Alpha Gene for Conjugal Transfer of DNA from *Escherichia coli* to Gram-Positive Bacteria. Gene.

[46] Takamatsu D., Osaki M., Sekizaki T. (2001). Thermosensitive Suicide Vectors for Gene Replacement in *Streptococcus suis*. Plasmid.

[47] Zhang T., Ding Y., Li T., Wan Y., Li W., Chen H., Zhou R. (2012). A Fur-like protein PerR regulates Two Oxidative Stress Response Related Operons *dpr* and *metQIN* in *Streptococcus suis*. BMC Microbiol..

[48] Dortet L., Mostowy S., Samba-Louaka A., Gouin E., Nahori M.A., Wiemer E.A., Dussurget O., Cossart P. (2011). Recruitment of the Major Vault Protein by InlK: A Listeria Monocytogenes Strategy to Avoid Autophagy. PLoS Pathog..

[49] Liu W., Tan M., Zhang C., Xu Z., Li L., Zhou R. (2018). Functional Characterization of *murB*-*potABCD* Operon for Polyamine Uptake and Peptidoglycan Synthesis in *Streptococcus suis*. Microbiol. Res..

[50] Tan M.-F., Gao T., Liu W.-Q., Zhang C.-Y., Yang X., Zhu J.-W., Teng M.-Y., Li L., Zhou R. (2015). MsmK, an ATPase, Contributes to Utilization of Multiple Carbohydrates and Host Colonization of *Streptococcus suis*. PLoS ONE.

[51] Li W., Hu X., Liu L., Chen H., Zhou R. (2011). Induction of Protective Immune Response against *Streptococcus suis* serotype 2 infection by the Surface Antigen HP0245. FEMS Microbiol. Lett..

[52] Zheng C., Xu J., Li J., Hu L., Xia J., Fan J., Guo W., Chen H., Bei W. (2014). Two Spx Regulators Modulate Stress Tolerance and Virulence in *Streptococcus suis* Serotype 2. PLoS ONE.

[53] Wang Y., Zhang W., Wu Z., Lu C. (2011). Reduced Virulence is an Important Characteristic of Biofilm Infection of *Streptococcus suis*. FEMS Microbiol. Lett..

[54] Kuru E., Tekkam S., Hall E.K., Brun Y.V., Van Nieuwenhze M.S. (2015). Synthesis of Fluorescent D-amino Acids and their use for Probing Peptidoglycan Synthesis and Bacterial Growth in Situ. Nat. Protoc..

[55] Lin L., Xu L., Lv W., Han L., Xiang Y., Fu L., Jin M., Zhou R., Chen H., Zhang A. (2019). An NLRP3 Inflammasome-Triggered Cytokine Storm Contributes to Streptococcal Toxic Shock-Like Syndrome (STSLS). PLoS Pathog..

[56] Pensinger D.A., Schaenzer A.J., Sauer J.-D. (2018). Do Shoot the Messenger: PASTA Kinases as Virulence Determinants and Antibiotic Targets. Trends Microbiol..

[57] Huerta-Cepas J., Szklarczyk D., Heller D., Hernández-Plaza A., Forslund S.K., Cook H.V., Mende D.R., Letunic I., Rattei T., Jensen L.J. (2019). eggNOG 5.0: A Hierarchical, Functionally and Phylogenetically Annotated Orthology Resource based on 5090 Organisms and 2502 Viruses. Nucleic Acids Res..

[58] Zheng F., Ji H., Cao M., Wang C., Feng Y., Li M., Pan X., Wang J., Qin Y., Hu F. (2011). Contribution of the Rgg Transcription Regulator to Metabolism and Virulence of *Streptococcus suis* Serotype 2. Infect. Immun..

[59] Deutscher J., Aké F.M.D., Derkaoui M., Zébré A.C., Cao T.N., Bouraoui H., Kentache T., Mokhtari A., Milohanic E., Joyet P. (2014). The Bacterial Phosphoenolpyruvate: Carbohydrate Phosphotransferase System: Regulation by Protein Phosphorylation and Phosphorylation-Dependent Protein-Protein Interactions. Microbiol. Mol. Biol. Rev..

[60] Macek B., Forchhammer K., Hardouin J., Weber-Ban E., Grangeasse C., Mijakovic I. (2019). Protein Post-Translational Modifications in Bacteria. Nat. Rev. Microbiol..

[61] Grangeasse C. (2016). Rewiring the Pneumococcal Cell Cycle with Serine/Threonine- and Tyrosine-kinases. Trends Microbiol..

[62] Fleurie A., Manuse S., Zhao C., Campo N., Cluzel C., Lavergne J.-P., Freton C., Combet C., Guiral S., Soufi B. (2014). Interplay of the Serine/Threonine-Kinase StkP and the Paralogs DivIVA and GpsB in Pneumococcal Cell Elongation and Division. PLoS Genet..

[63] Agarwal S., Pancholi P., Pancholi V. (2011). Role of Serine/Threonine Phosphatase (SP-STP) in *Streptococcus pyogenes* Physiology and Virulence*. J. Biol. Chem..

[64] Piñas G.E., Vizcaino N.R., Barahona N.Y.Y., Cortes P.R., Durán R., Badapanda C., Rathore A., Bichara D.R., Cian M.B., Olivero N.B. (2018). Crosstalk between the Serine/Threonine Kinase StkP and the Response Regulator ComE controls the Stress Response and Intracellular Survival of *Streptococcus pneumoniae*. PLoS Pathog..

[65] Libby E.A., Reuveni S., Dworkin J. (2019). Multisite Phosphorylation Drives Phenotypic Variation in (p)ppGpp Synthetase-Dependent Antibiotic Tolerance. Nat. Commun..

[66] Monteiro J.M., Pereira A.R., Reichmann N.T., Saraiva B.M., Fernandes P.B., Veiga H., Tavares A.C., Santos M., Ferreira M.T., Macario V. (2018). Peptidoglycan Synthesis Drives an FtsZ-Treadmilling-Independent Step of Cytokinesis. Nature.

[67] Lutkenhaus J. (2007). Assembly Dynamics of the Bacterial MinCDE System and Spatial Regulation of the Z Ring. Annu. Rev. Biochem..

[68] Sacco E., Cortes M., Josseaume N., Rice L.B., Mainardi J.-L., Arthur M. (2014). Serine/Threonine Protein Phosphatase-Mediated Control of the Peptidoglycan Cross-Linking l, d-Transpeptidase Pathway in *Enterococcus faecium*. mBio.

[69] Hsu P.-C., Chen C.-S., Wang S., Hashimoto M., Huang W.-C., Teng C.-H. (2020). Identification of MltG as a Prc Protease Substrate Whose Dysregulation Contributes to the Conditional Growth Defect of Prc-Deficient *Escherichia coli*. Front. Microbiol..

[70] Zheng J.J., Perez A.J., Tsui H.-C.T., Massidda O., Winkler M.E. (2017). Absence of the KhpA and KhpB (JAG/EloR) RNA-binding Proteins Suppresses the Requirement for PBP2b by Overproduction of FtsA in *Streptococcus pneumoniae* D39. Mol. Microbiol..

[71] Patel V., Wu Q., Chandrangsu P., Helmann J.D. (2018). A Metabolic Checkpoint Protein GlmR is important for Diverting Carbon into Peptidoglycan biosynthesis in *Bacillus subtilis*. PLoS Genet..

[72] Rued B.E., Zheng J.J., Mura A., Tsui H.T., Boersma M.J., Mazny J.L., Corona F., Perez A.J., Fadda D., Doubravova L. (2017). Suppression and Synthetic-Lethal Genetic Relationships of DeltagpsB mutations indicate that GpsB Mediates Protein Phosphorylation and Penicillin-Binding Protein Interactions in *Streptococcus pneumoniae* D39. Mol. Microbiol..

[73] Ulrych A., Holečková N., Goldová J., Doubravová L., Benada O., Kofroňová O., Halada P., Branny P. (2016). Characterization of Pneumococcal Ser/Thr Protein Phosphatase phpP Mutant and Identification of a Novel PhpP Substrate, Putative RNA Binding Protein Jag. BMC Microbiol..

[74] McAvoy T., Nairn A.C. (2010). Serine/Threonine Protein Phosphatase Assays. Curr. Protoc. Mol. Biol..

