# Supplementary material for: Comparative Phenotypic, Proteomic, and Phosphoproteomic Analysis Reveals Different Roles of Serine/Threonine Phosphatase and Kinase in the Growth, Cell Division, and Pathogenicity of Streptococcus suis
Source: Microorganisms. 2021 Nov 26;9(12):2442. doi: 10.3390/microorganisms9122442 (PMC8707513; doi:10.3390/microorganisms9122442)
Supplement: Supplementary file 1 [file microorganisms-09-02442-s001.zip › Supplementary Figures.pdf]

Supplementary Figures

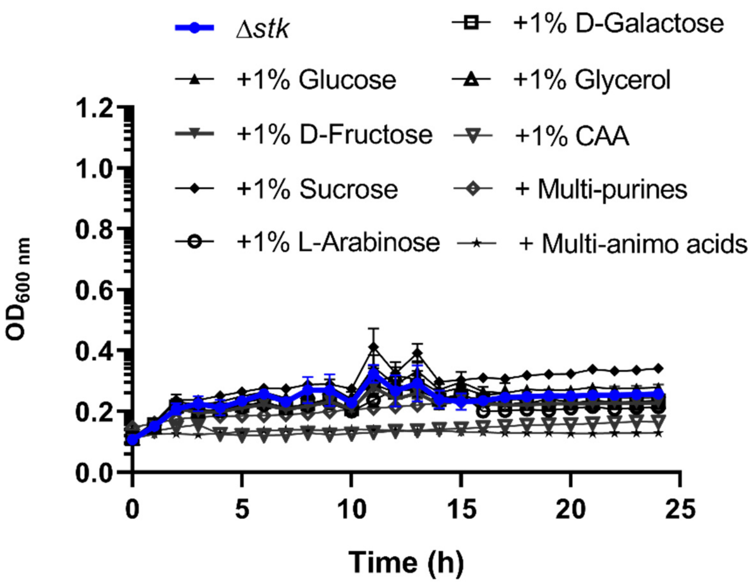

Figure S1. Growth analysis of Δ*stk* strains. (A) The growth of Δ*stk* strain in CDM or CDM supplemented with each indicated nutrient. Growth was monitored with an automatic plate reader with shaking at 37°C. Data are presented as means ± SD of triplicate.

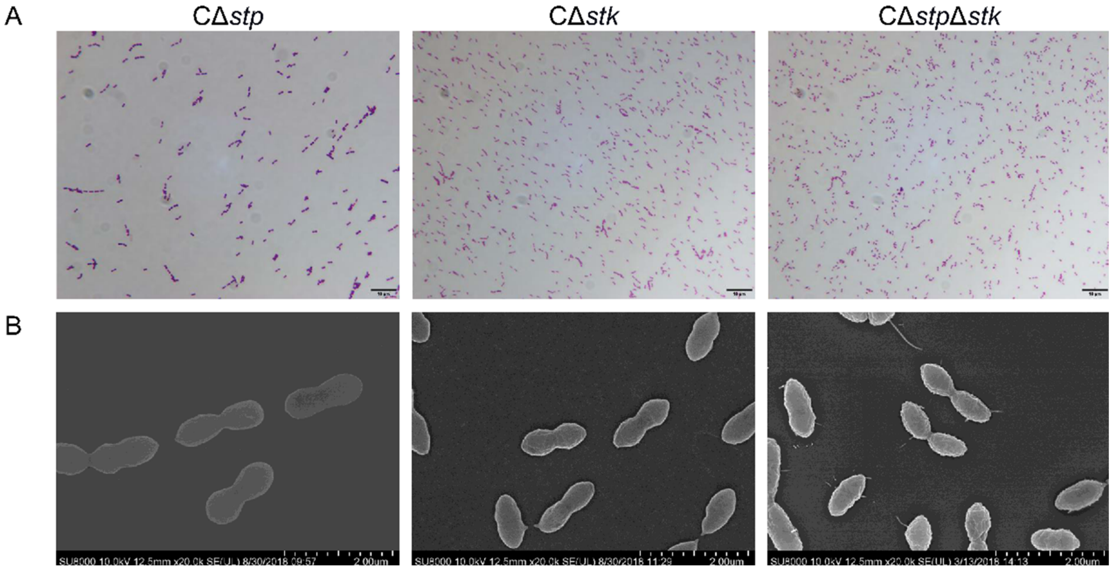

Figure S2. Morphological analysis of CΔ*stp*, CΔ*stk*, and CΔ*stp*Δ*stk* strains. Gram staining (A) and scanning electron microscopy (SEM) (B) analysis of CΔ*stp*, CΔ*stk*, and CΔ*stp*Δ*stk* strains.

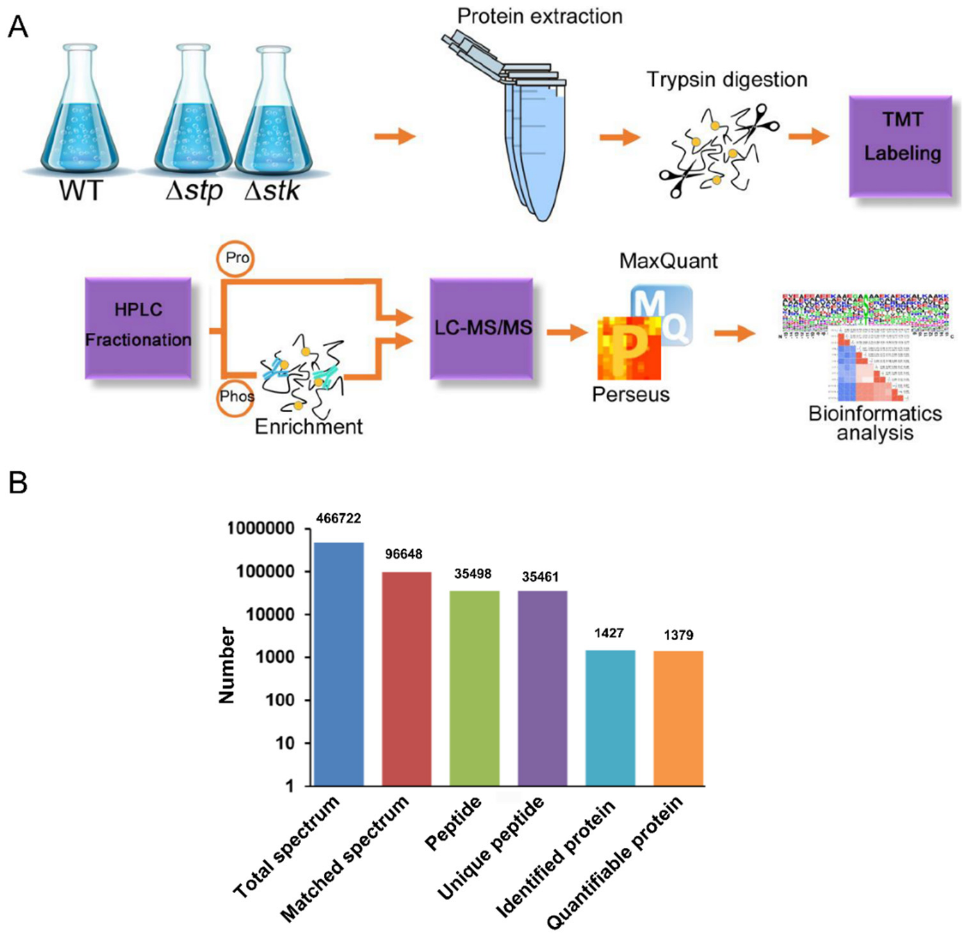

Figure S3. Comparative proteomic analysis between the WT and Δ*stp* or Δ*stk* strains. (A) The systematic workflow of the quantitative profiling of the global mass spectrum. (B) The basic statistics of mass spectrum data.

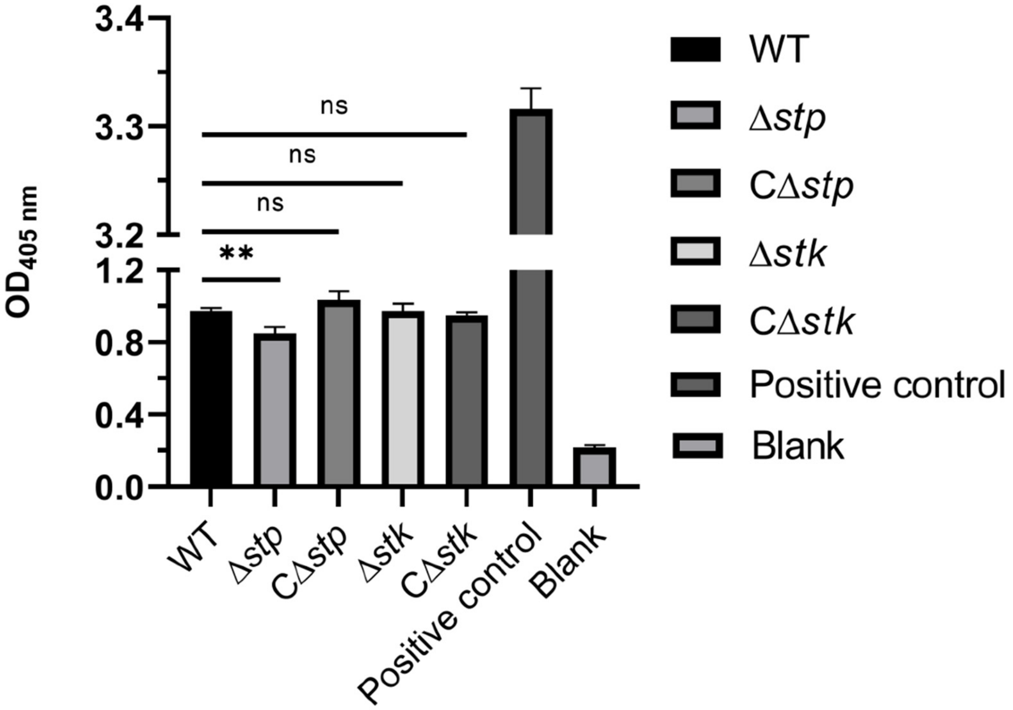

Figure S4. Phosphatase activity assay. Cells at the mid-log phase of each indicated strain were collected, washed three times with buffer1 [20 mM Tris-HCl (pH 8.0) and 200 mM $NaCl_2$], resuspended in lyse buffer [50 mM Tris-HCl (pH 8.0), 150 mM $NaCl_2$, 100 U/mL mutanolysin (Sigma), and 1 mM PMSF], and lysed by sonication. The amount of total protein loaded was normalized using a Micro BCA protein assay kit (Cwbiotech, China). Samples were mixed with buffer2 [50 mM Tris-HCl (pH 8.0), 2 mM $MnCl_2$, and 20 mM p-nitrophenyl phosphate (p-NPP)], and incubated at 37℃ for 10 min. The absorbance at 405 nm was then measured. Purified recombinant STP protein was used as the positive control, the reaction buffer2 used as the blank. Data are presented as means ± SD of triplicate. Statistical significance was determined by two-tailed, unpaired Student's *t*-tests (ns, p value > 0.05; *, p value < 0.05).
