# Supplementary material for: Comparative Phenotypic, Proteomic, and Phosphoproteomic Analysis Reveals Different Roles of Serine/Threonine Phosphatase and Kinase in the Growth, Cell Division, and Pathogenicity of Streptococcus suis
Source: Microorganisms. 2021 Nov 26;9(12):2442. doi: 10.3390/microorganisms9122442 (PMC8707513; doi:10.3390/microorganisms9122442)
Supplement: Supplementary file 1 [file microorganisms-09-02442-s001.zip › Table S1.pdf]

Table S1. Strains and plasmids used in this study.

Strain or plasmid	Genotype or characteristics*	Reference or source
***S. suis* strains**		
SC19	*S. suis* serotype 2 isolate, wild-type strain (Strep^r)	[1]
Δ*stp*	SC19 with CDS of *stp* replaced by the CDS of Erm^r (Strep^r Erm^r)	This study
Δ*stk*	SC19 with *stk* deleted (Strep^r)	This study
Δ*stp*Δ*stk*	SC19 with *stp* and *stk* deleted (Strep^r)	This study
CΔ*stp*	Complemented strain of Δ*stp* by integrating *stp* into the chromosome (Strep^r)	This study
CΔ*stk*	Complemented strain of Δ*stk* by integrating *stk* into chromosome (Strep^r)	This study
CΔ*stp*Δ*stk*	Complemented strain of Δ*stp*Δ*stk* by integrating *stp*/*stk* into the chromosome (Strep^r)	This study
**Plasmids**		
pAT18	A plasmid carrying an erythromycin resistance gene (Erm^r)	[2]
pSET4s	Temperature-sensitive *E. coli*-*S. suis* shuttle vector (Spc^r)	[3]
pSET4s-P	Derived from pSET4s for deletion of *stp* in SC19 (Spc^r)	This study
pSET4s-K	Derived from pSET4s for deletion of *stk* in SC19 (Spc^r)	This study
pSET4s-PK	Derived from pSET4s for deleting *stp*/*stk* in SC19 (Spc^r)	This study

* Strep^r, streptomycin resistant; Erm^r, erythromycin resistant; Spc^r, spectinomycin resistant.

References:

1. Li, W.; Liu, L.; Chen, H.; Zhou, R., Identification of Streptococcus suis genes preferentially expressed under iron starvation by selective capture of transcribed sequences. *FEMS Microbiol Lett* **2009,** 292, (1), 123-33.
2. Trieu-Cuot, P.; Carlier, C.; Poyart-Salmeron, C.; Courvalin, P., Shuttle vectors containing a multiple cloning site and a lacZ alpha gene for conjugal transfer of DNA from Escherichia coli to gram-positive bacteria. *Gene* **1991,** 102, (1), 99-104.
3. Takamatsu, D.; Osaki, M.; Sekizaki, T., Thermosensitive suicide vectors for gene replacement in Streptococcus suis. *Plasmid* **2001,** 46, (2), 140-8.
