# Supplementary material for: Comparative Phenotypic, Proteomic, and Phosphoproteomic Analysis Reveals Different Roles of Serine/Threonine Phosphatase and Kinase in the Growth, Cell Division, and Pathogenicity of Streptococcus suis
Source: Microorganisms. 2021 Nov 26;9(12):2442. doi: 10.3390/microorganisms9122442 (PMC8707513; doi:10.3390/microorganisms9122442)
Supplement: Supplementary file 1 [file microorganisms-09-02442-s001.zip › Table S2.pdf]

Table S2. oligonucleotides used in this study

Primer name	Sequence (5′—3′)*
Pup_F	CCTGCAGGTCGACTCTAGACAGTTGTCAATGAGGCAGTT
Pup_R	AATATTTTATATTTTTGTTCATGTTGTTCGAACGTTTCTGTC
Erm_F	GACAGAAACGTTCGAACAACATGAACAAAAATATAAAATATT
Erm_R	AACCTACTGTAATATTATCTTTATTTCCTCCCGTTAAATA
Pdown_F	TATTTAACGGGAGGAAATAAAGATAATATTACAGTAGGTT
Pdown_R	TTGTAAAACGACGGCCAGTTCAAGACTTTGTAGCGTGTA
Kup_F	TGTAAAACGACGGCCAGTGAATTCTGCCAAAGCTTGAAAATTGG
Kup_R	CTCCCCAACCTTATTCATCTTACCTAGCCTCCTCCGTAAT
Kdown-F	ATTACGGAGGAGGCTAGGTAAGATGAATAAGGTTGGGGAG
Kdown-R	CTGCAGGTCGACTCTAGAGGATCCAATATGATTCACCCAGGAAA
PKup-F	GTAAAACGACGGCCAGTGAATTCAAATTTGTTAATGCCAT
PKup-R	ACCCCAACCTTATTCATCTAATTTTTATCGTTGCTAAGC
PKdown-F	GCTTAGCAACGATAAAAATTAGATGAATAAGGTTGGGGA
PKdown-R	CTGCAGGTCGACTCTAGAGGATCCAATATGATTCACCCAGGAA
Psmup_F	CCTGCAGGTCGACTCTAGACAGTTGTCAATGAGGCAGTT
Psmup_R	ATCTGTTGCTGCCATCTCGCTAGCAATATGGCCGGCACG
Psmdown_F	CGAGATGGCAGCAACAGATTTGGGAGCTGCTTGGGTTGA
Psmdown_R	TTGTAAAACGACGGCCAGTTCAAGACTTTGTAGCGTGTA
Ksmup_F	CCTGCAGGTCGACTCTAGATGCCAAAGCTTGAAAATTGG
Ksmup_R	ACTGTTTGTATGCGGTACCTTCGTTTCAACATTGGCTTG
Ksmdown_F	GGTACCGCATACAAACAGTTCTGCTCAAGTTTCTGCGAC
Ksmdown_R	TTGTAAAACGACGGCCAGTAATATGATTCACCCAGGAAA
PKsmup_F	CCTGCAGGTCGACTCTAGAGCGTAAGCTTAGTCAGGATT
P_F	GCTGATGGAATGGGTGGTC
P_R	TGCAACCATCCATTCACGGA
K_F	CATAGCGGCTGGACTCATG
K_R	CTACAGTCGCTGTCGCTTC
*gapdH*_F	TGGTTCTTGACGGACCACAC
*gapdH*_R	CAGCACCGTCAAGTTTACCG
